# Modeling, validation and verification of three-dimensional cell-scaffold contacts from terabyte-sized images

**DOI:** 10.1186/s12859-017-1928-x

**Published:** 2017-11-28

**Authors:** Peter Bajcsy, Soweon Yoon, Stephen J. Florczyk, Nathan A. Hotaling, Mylene Simon, Piotr M. Szczypinski, Nicholas J. Schaub, Carl G. Simon, Mary Brady, Ram D. Sriram

**Affiliations:** 1 Information Technology Laboratory, National Institute of Standards and Technology, Gaithersburg, MD USA; 2 Material Measurement Laboratory, National Institute of Standards and Technology, Gaithersburg, MD USA; 3 Lodz University of Technology, Łódź, Poland; 4 Department of Materials Science & Engineering, University of Central Florida, Orlando, FL USA; 5 National Eye Institute, National Institute of Health, Bethesda, MD USA; 6 Dakota Consulting Inc, Silver Spring, MD USA

**Keywords:** Co-localization, Cellular measurements, Cell-scaffold contact, Segmentation models, Contact evaluation, Web-based verification, Large-volume 3D image processing

## Abstract

**Background:**

Cell-scaffold contact measurements are derived from pairs of co-registered volumetric fluorescent confocal laser scanning microscopy (CLSM) images (z-stacks) of stained cells and three types of scaffolds (i.e., spun coat, large microfiber, and medium microfiber). Our analysis of the acquired terabyte-sized collection is motivated by the need to understand the nature of the shape dimensionality (1D vs 2D vs 3D) of cell-scaffold interactions relevant to tissue engineers that grow cells on biomaterial scaffolds.

**Results:**

We designed five statistical and three geometrical contact models, and then down-selected them to one from each category using a validation approach based on physically orthogonal measurements to CLSM. The two selected models were applied to 414 z-stacks with three scaffold types and all contact results were visually verified. A planar geometrical model for the spun coat scaffold type was validated from atomic force microscopy images by computing surface roughness of 52.35 nm ±31.76 nm which was 2 to 8 times smaller than the CLSM resolution. A cylindrical model for fiber scaffolds was validated from multi-view 2D scanning electron microscopy (SEM) images. The fiber scaffold segmentation error was assessed by comparing fiber diameters from SEM and CLSM to be between 0.46% to 3.8% of the SEM reference values. For contact verification, we constructed a web-based visual verification system with 414 pairs of images with cells and their segmentation results, and with 4968 movies with animated cell, scaffold, and contact overlays. Based on visual verification by three experts, we report the accuracy of cell segmentation to be 96.4% with 94.3% precision, and the accuracy of cell-scaffold contact for a statistical model to be 62.6% with 76.7% precision and for a geometrical model to be 93.5% with 87.6% precision.

**Conclusions:**

The novelty of our approach lies in (1) representing cell-scaffold contact sites with statistical intensity and geometrical shape models, (2) designing a methodology for validating 3D geometrical contact models and (3) devising a mechanism for visual verification of hundreds of 3D measurements. The raw and processed data are publicly available from https://isg.nist.gov/deepzoomweb/data/ together with the web -based verification system.

**Electronic supplementary material:**

The online version of this article (doi:10.1186/s12859-017-1928-x) contains supplementary material, which is available to authorized users.

## Background

The problem of 3D contact measurements between a cell and its surrounding scaffold is related to co-localization of two objects from dual-color fluorescent microscopy z-stacks [1–4] where each channel is imaged to excite either cell or scaffold stain. The z-stacks are 3D images formed by a set of uniformly-spaced cross-sectional 2D images along a z-axis. In general, co-localization refers to the spatial overlap of at least two fluorescent labels (staining dyes) emitting distinct wavelengths. The mathematical definition of the spatial overlap in volumetric data (z-stacks) can be viewed as a co-occurrence of two labels at the same or neighboring locations, or as a correlation of intensities at the co-occurring locations. In this work, we use the term 3D contact to refer to the co-occurrence of fluorescent labels because of our interest in measuring the shape of cell-scaffold spatial interactions.

**Fig. 1:**
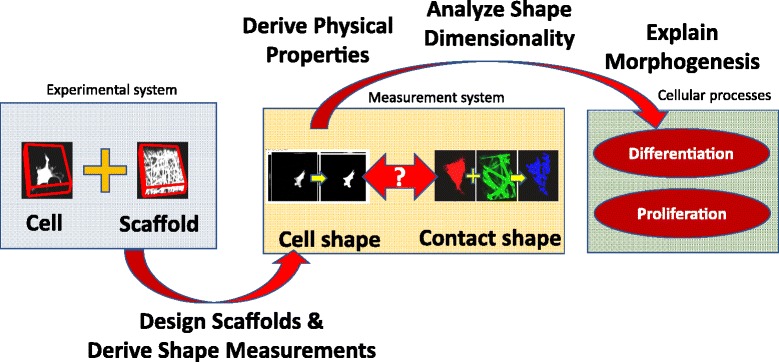
Biological motivation behind the cell-scaffold contact measurements

The shape measurements of cell-scaffold contacts are important for tissue engineers that grow cells on a variety of biomaterial scaffolds. One of the many challenges in growing cells is to discover how cellular processes (for instance, differentiation and proliferation) and cell shape changes are coordinated during morphogenesis [5]. In the past, it has been reported that (1) a type of scaffold drives the cell shape [6, 7], and (2) scaffold substrate effects on the shape of human bone marrow stromal cells (hBMSCs) can influence their behavior and differentiation [8–11]. However, there is a lack of understanding of the relationship between cell shape and cell-scaffold contact shape, and how these measurements may serve as predictors of cell differentiation fate. The biological motivation is illustrated in Fig. 1.

Current approaches to designing 3D scaffold niches focus on assessing the effect of a design for a desirable cell function, such as proliferation, expansion, or differentiation towards a target lineage. Although this approach is very useful, it does not enable a reasoned approach to scaffold design where the scaffold is constructed to drive the cells into a particular morphology that will preferentially guide the cells towards the desired function. Although there is extensive evidence linking cell shape and function [10, 12–14], there is a lack of quantitative data regarding the 3D morphology of cell-material interactions in biomaterial scaffolds. In order to address these issues, the 3D shape of contacts between scaffolds and primary human bone marrow stromal cells (hBMSCs) was quantitatively evaluated in three biomaterial substrates made from poly(lactic-co-glycolic acid) (PLGA): Spun Coat (SC), Medium Microfibers (MMF) and Large Microfibers (MF). hBMSCs were used for this study because of their clinical relevance to tissue engineering and regenerative medicine [15] and due to the intense interest in guiding their behavior through environmental cues [12, 16–22]. The three chosen substrates make an interesting system to study because fibrous scaffolds (MF and MMF) have been observed to drive osteogenic differentiation of hBMSCs while the flat substrates did not [9, 11, 22]. By constructing all three substrates from the same material (PLGA), the effect of substrate structure could be studied in the absence of changes in composition. A 24 h cell culture time point was selected for imaging to give the cells enough time to achieve a stable morphology but not so much time that the cells had proliferated or differentiated.

Although tissue engineers aim to improve scaffold design in order to guide cell behavior, the role of the geometry of cell-scaffold contacts has not been adequately considered. Cell shape is dictated by the geometry of cell matrix contacts as the cell can only spread and adhere to the matrix which surrounds it. In addition, cell-adhesion sites, often described as focal adhesions, may trigger signaling events that guide gene expression and cell behavior. Thus, the geometry and spacing of cell adhesion sites will influence gradients and timing of these signaling events. For these reasons, tissue engineers can benefit from 3D mapping of cell-scaffold contact sites in order to generate new insights for designing scaffolds that guide cell function. For example, cell shape alone might not convey information about cell-scaffold contact surface for cells residing on hydrophobic versus hydrophilic scaffolds with the same geometry. Nevertheless, such contact measurements have not been acquired due to the complexity of these measurements as they require information about both the cell and the scaffold. Our motivation for the work comes from the need to design a measurement methodology for cell-scaffold contact sites so that cell differentiation fate can be reliably predicted.

**Fig. 2:**
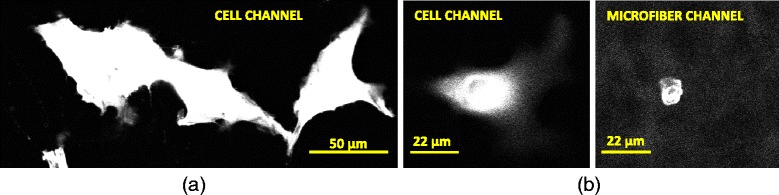
Examples of one field of view with (**a**) multiple cells in proximity and (**b**) bleed-through from cell channel to microfiber channel

Several challenges of measuring cell-scaffold contact shapes can be summarized as follows:

1. Our insufficient knowledge about the spatial and intensity statistics as well as geometry of foreground objects (cell membrane, scaffold) limits our ability to detect foreground reliably (see Fig. 2a).
2. The difficulties in acquiring orthogonal cell-scaffold contact measurements and validating automated analytical algorithms constrain our measurement confidence (i.e., orthogonal measurements refer to those physics-based methods that are based on other than fluorescent imaging modality).
3. Large RAM (random access memory) requirements (≈3 GB just to load the pair of input z-stacks) and large data volume (>1 TB) impose computational and execution time burden on the analyses.
4. Fluorescent staining dyes emit light at overlapping wavelength ranges which introduces intensity bleed-through across cell and scaffold co-registered z-stacks (see Fig. 2b). This leads to a bias in co-localization (i.e., locations of a stained cell have higher intensity values in a scaffold channel than background and vice versa).
5. Design of an efficient and geographically accessible visual verification system of complicated 3D contact shapes over several hundreds of z-stacks is difficult.

The specific problem addressed in this work can be formulated as a design of a measurement methodology for cell-scaffold contacts over terabyte-sized collections of dual-color fluorescent confocal microscopy z-stacks. Following the past work [7], the measurement methodology consists of three components:

1. Modeling of (a) an object of interest (cell or scaffold) in each z-stack for foreground segmentation and (b) a cell-scaffold contact based on the relative spatial positions of the segmented objects.
2. Validation of the accuracy of segmentation and contact models.
3. Verification of several hundreds of automatically detected cell-scaffold contacts through visual inspection.

**Fig. 3:**
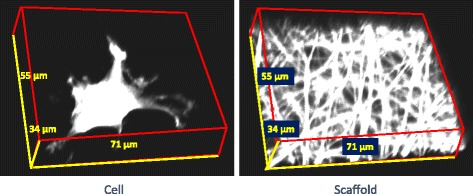
A pair of cell and scaffold z-stacks for the microfiber scaffold type

The experimental design includes three types of scaffolds (SCMFMMF), eight cell-scaffold contact methods, and three human experts performing verification. Figure 3 shows one example of a pair of cell and scaffold z-stacks. These three scaffolds represent geometries that cause cells to have contacts with scaffolds at one or multiple z-planes (SP – one contact plane, MF and medium MMF – larger than 3 contact planes).

We approach the design problem for the three-component measurement methodology by addressing challenges specific to each component as summarized in Table 1. The modeling challenges related to our insufficient knowledge about statistics and geometry of foreground objects are approached as an optimization problem over a set of statistical and geometrical models. The modeling challenges also include large RAM and execution time requirements due to the terabyte-sized data collection. To alleviate these challenges, the regions of interests (ROIs) are cropped from each image z-stack such that all cells and their surrounding scaffold are included. The validation challenges related to the difficulties in acquiring physics-based orthogonal cell-scaffold contact measurements are addressed by using Scanning Electron Microscopy (SEM) and Atomic Force Microscopy (AFM). Finally, the verification challenges related to raw and processed data quality due to complicated 3D contact shapes are handled by designing a web-based visual inspection system that accommodates verification time and accuracy tradeoffs.

**Table 1:** Summary of problems in cell-scaffold contact measurements and our approaches

Challenges	Approach
How to model cell, scaffold, and cell-scaffold contact from two-channel z-stacks?	Design statistical and geometrical models for segmentation
	Use the law of total probability in statistical models, and geometrical intersections in geometrical models for cell-scaffold contact detection
How to validate geometrical cell-scaffold contact models and assess the accuracy of all contact models?	Validate cylindrical and planar geometrical models using multi-view SEM and AFM measurements
	Assess the accuracy of all models applied to CLSM z-stacks by comparing single fiber radius measurements derived from CLSM z-stacks against the reference values extracted from SEM images
How to handle RAM and processing time requirements for TB-sized z-stack collection?	Reduce the amount of data to be processed by cropping ROIs defined by cell bounding boxes and reduce the processing time by utilizing parallel processing
How to verify quality of 3D contact measurements over several hundreds of [cell, scaffold] pairs of z-stacks?	Design a web system for visual verification using three orthogonal max 2D projections of cell and its segmentation, and six movies combining dynamic rotations of contacts and information layers of 3D cell-scaffold-contact per [cell, scaffold] pair

Our work on modeling, validation and verification can be related to the published methods that focus on the problems of co-localization, foreground modeling, 3D segmentation validation, and verification of 3D contacts at large scale. The past work is summarized in Table 2 together with the relationship to our presented work. Detailed descriptions of related work can be found in Additional file 1.

**Table 2:** Relationship of past work to our approach. The abbreviations of imaging modalities refer to scanning electron microscopy (SEM), confocal laser scanning microscopy (CLSM), X-ray micro-computed tomography (μCT), and selective plane illumination microscopy (mSPIM)

Past Work	Relationship to our approach
Co-localization detection	Co-localization detection
• Spatial image cross-correlation spectroscopy (ICCS) [1–4] - Pearson, Spearman’s rank.	• ICCS is not applicable since it does not capture spatial information.
	• Replaced manual segmentation with automated object-based analysis.
• Object-based analysis [35] with manual segmentation.	
Foreground modeling	Foreground modeling
• Statistical: scatterplot of two channel intensities [4] with a single model.	• Statistical: used scatterplot with optimization over multiple statistical models.
• Geometrical: fiber segmentation based on many software packages including IvanTK, NeuronJ, Simple Neurite Tracer, Vaa3D, and Vascular Modelling Toolkit (VMTK).	• Geometrical: could not use existing software designed for vascular or brain structures (not fiber scaffolds), and some software worked only in 2D and required manual identification of end points.
Validation of 3D Segmentation	Validation of 3D Segmentation
• Manual reference [36–41].	• Manual reference is hard to create for 3D objects.
• Orthogonal measurements using μCT, SEM and CLSM [42], and mSPIM [43].	• Used orthogonal measurements of a single fiber imaged via multi-view 2D SEM and 3D CLSM.
Visual verification of 3D contacts at large scale	Visual verification of 3D contacts at large scale
• Not aware of any previous work.	• We designed a web system with three orthogonal max projections and 6 animated movies per contact.

Based on the reviewed related work, the novelty and contributions of our work come from:

1. creating and optimizing cell-scaffold contact representations that incorporate five statistical and three geometrical models,
2. designing a methodology for validating fiber segmentation using reference SEM and fluorescent confocal measurements of single fibers, and
3. devising a mechanism for rapid visual verification of hundreds of 3D measurements.

An additional contribution comes from the fact that we created the largest collection of 3D cell-scaffold measurements in the bio-manufacturing community. The data are available at https://isg.nist.gov/deepzoomweb/data and the web-based verification system for cell segmentation and cell-scaffold contacts is available at https://isg.nist.gov/CellScaffoldContact/app/index.html.

The main manuscript presents materials and methods, experimental results, discussion of quantitative and qualitative results, and conclusions. The appendices contain the detailed description of related work (Additional file 1), cell segmentation algorithm (Additional file 2), model for cropping contact regions of interest (Additional file 3), statistical model of background (Additional file 4), statistical models for segmenting all scaffold types (Additional file 5), algorithms based on statistical models for segmenting all scaffold types (Additional file 6), algorithm based on planar geometrical model for segmenting spun coat scaffolds (Additional file 7), algorithms based on cylindrical geometrical models for segmenting fiber scaffolds (Additional file 8), evaluations of goodness-to-fit for planar model used for modeling spun-coat scaffolds (Additional file 9), and validation steps based on 2D SEM and 3D CLSM data of single fibers (Additional file 10).

## Methods

Although we focus on shape metrology, one could view the materials and methods as a foundation for answering a question: “How would cell, scaffold, and cell-scaffold interaction shape characteristics affect cell fate (differentiation and proliferation)?” Answering this and other related questions is the driving factor behind the next sections.

### Materials

The materials and digital data are divided into a set supporting cell-scaffold contact measurements and a set acquired for algorithmic validation purposes.

#### Cell-scaffold contact measurements

In this paper, the data acquisition focuses on the measurements establishing the effect of scaffold types on cell morphology and on cell behavior. The data sets are acquired by CLSM as images (z-stacks) of cells cultured on three different scaffolds. The three scaffolds are described in Table 3.

**Table 3:** Scaffold type abbreviations and descriptions

Scaffold Name and Abbreviation	Scaffold Material Description
Spun Coat (SC)	Flat films of spun-coat Poly lactic-co-glycolic acid (PLGA)
Large Microfibers (MF)	Electrospun PLGA microfibers (diameter equal to 2.6 μm)
Medium Microfibers (MMF)	Electrospun PLGA microfibers (diameter equal to 1.1 μm)

##### Cell preparation

Primary human bone marrow stromal cells (hBMSCs, Tulane Center for Gene Therapy, donor #8004 L, 22 yr. male, iliac crest) were cultured in medium (α-MEM containing 16.5% by vol. fetal bovine serum, 4 mmol/L L-glutamine, and 100 units/mL penicillin and 100 μg/mL streptomycin) in a humidified incubator (37 °C with 5% CO2 by vol.) to 70% confluency, trypsinized (0.25% trypsin by mass containing 1 mmol/L ethylenediaminetetraacetate (EDTA), Invitrogen) and seeded onto substrates (scaffolds) at passage 4. SC, MF and MMF substrates (see Table 3) were placed in multi-well plates and cells suspended in medium were seeded onto them at a density of 1250 cells/cm^2^. hBMSCs were cultured for 1 day for all treatments prior to imaging. After 1 day, culture, cells on scaffolds were fixed with 3.7% (vol./vol.) formaldehyde and stained for cell membrane (5 μmol/L Oregon-Green maleimide, Life Technologies) and nucleus (0.03 mmol/L 4′,6-diamidino-2-phenylindole, DAPI, Life Technologies). More than 100 cells were imaged per scaffold type to provide statistically meaningful results.

##### Scaffold preparation

The MMF and MF scaffolds for cell culture were created by electrospinning a blend of two types of poly(lactic-co-glycolic acid) (PLGA): using the same polymer mixture for the MMF and MF treatments. The polymer mixture was 90% mass fraction PLGA Poly lactic-co-glycolic acid (PLGA 50:50 M ratio of L to G, relative molecular mass ≈110,000 g/mol, Lactel Absorbable Polymers) and 10% mass fraction PLGA-Flamma Fluor FKR648 (PLGA 50:50 M ratio of L to G, relative molecular mass ≈25,000 g/mol, Flamma Fluor FKR648 ester- linked to the PLGA, Akina Inc., Polyscitech). Flamma Fluor FKR648 was covalently bound to the PLGA via an ester linkage to prevent leaching into the cell culture medium. For MF scaffolds, the PLGA/PLGA-FKR648 blend was dissolved in 3:1 acetone: ethyl acetate and electrospun (18 gauge steel needle, 2.3 ml/h, tip to collector distance of 15 cm, aluminum foil target) at 14 kV (high voltage generator, ES30P-5 W, Gamma High Voltage Research) to yield monodisperse PLGA nanofibers. For MMF scaffolds, the PLGA/PLGA-FKR648 blend was dissolved in acetone and electrospun (22 ga. steel needle, 1.25 mL/h, tip to collector distance of 15 cm, aluminum foil target) at 12 kV (high voltage generator, ES30P-5 W, Gamma High Voltage Research) to yield monodisperse PLGA nanofibers. For scanning electron microscope (SEM) imaging the PLGA mats were removed from the foil and cut into 5 mm × 5 mm squares.

##### Imaging

**Fig. 4:**
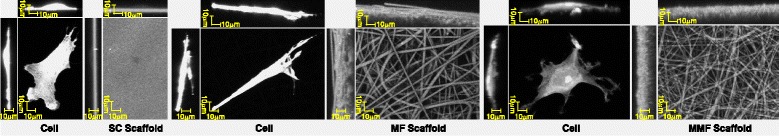
Cell and scaffold z-stack pairs for the three types of scaffolds (spun coat, microfibers, medium microfibers)

The samples were imaged with CLSM (Leica SP5 II, Leica Microsystems) using a 63× water-immersion objective (numerical aperture 0.9, 1 Airy unit). Prior to imaging, cell culture medium was removed and replaced with phosphate buffered saline (PBS) to reduce the background fluorescent signal. A z-stack with two channels was collected for each of 711 cells. The two channels corresponded to cell membrane (Oregon-Green - excitation 488 nm, emission 501 nm to 570 nm) and fiber scaffold (Flammafluor648 - excitation 633 nm, emission 652 nm to 708 nm). We also collected a single image of nucleus (DAPI - excitation 405 nm, emission 413 nm to 467 nm) to confirm that measured objects were cells (objects without a nucleus were discarded). Based on the manufacturer’s defined resolution for the 63× objective (XY = 217 nm and Z = 626 nm for 488 nm wavelength), we defined our acquisition fluorescent voxel dimensions in X, Y and Z respectively at 0.12 μm × 0.12 μm × 0.462 μm [2048 pixels × 2048 pixels in X and Y, up to 175 frames in Z]. Each z-frame in the z-stacks was exported as an 8 MB tif image with a resolution of 2048 pixels × 2048 pixels (246 μm × 246 μm) and 16 bits per pixel. Examples of z-frame tif images are shown in Fig. 4.

##### Data summary and quality assurance

The data collection initially generated z-stacks of 711 z-stacks of [cell, scaffold] pairs that were visually inspected. We kept only z-stacks with individual hBMSCs that were not touching other cells so that the contact measurements are per cell. Out of the initial 711 pairs, we eliminated 259 pairs due to out-of-stack cells (automated cell localization and focus failed) and 41 pairs due to very low background offset that would not allow us to estimate background intensity distribution model. After eliminating the total of 297 pairs, the remaining 411 [cell, scaffold] pairs were summarized in Table 4. Each z-stack was between 922.75 MB and 1468 MB [2048 pixels (X) × 2048 pixels (Y) × 110 to 175 pixels (Z)] which mapped to about 3 GB of RAM when a pair of [cell, scaffold] z-stacks was loaded.

**Table 4:** Summary of input z-stacks after initial quality control of 711 [cell, scaffold] pairs

Sample group	[cell, scaffold] pairs (z-stacks)	Image files (z-frames)	Size (GB)
SC	165	37,127	306
MF	135	47,927	400
MMF	114	40,176	337
Total	414	125,230	1043

#### Algorithmic model validation measurements

##### Surface roughness reference measurements of a spun coat scaffold to validate a planar geometrical contact model

Surface roughness of the SC films was measured using atomic force microscopy (AFM, Dimension Icon, Bruker, Billerica, MA). Six uniformly-distributed spots on a SC film sample were analyzed with each spot size of 50 μm × 50 μm (256 samples per scan line, 0.195 μm spatial resolution). The images were analyzed with Nanoscope Analysis (Bruker) and the root mean square (RMS) roughness was reported for each analyzed spot and averaged to produce a single value for the SC film.

##### Single fiber radius reference measurements to validate a cylindrical geometrical contact model and to assess accuracy of fiber scaffold segmentation

SEM was chosen to verify results from confocal epifluorescence mode (CLSM) because SEM is higher resolution than CLSM. However, SEM is conducted in the dry state, the CLSM was conducted under water immersion and PLGA fibers can swell when hydrated. To address this issue, the fibers that were imaged by SEM in the dry state were imaged by confocal via water immersion within 2 h of being immersed in PBS. Thus, swelling of the PLGA buffer should be minimal since it takes several days for PLGA to swell in buffer [23]**.**

**Fig. 5:**
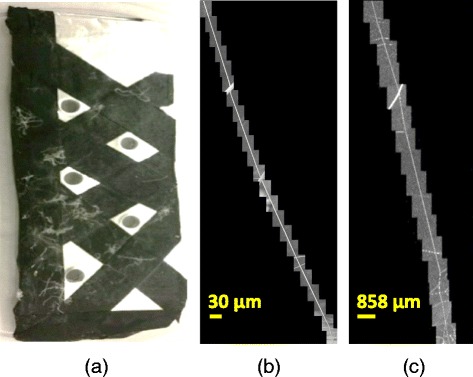
Single fiber data. (**a**) Single fibers mounted on a sample, (**b**) 2D SEM images stitched along one fiber, and (**c**) 2D max projections of 3D CLSM z-stacks that are roughly stitched along one fiber

We used the same polymer and spinning conditions (as indicated before) for the large microfiber (MF) sample. However, rather than spin the fibers onto an aluminum foil target, fibers were spun onto aluminum mounts. Aluminum mounts were 25 mm × 75 mm × 0.5 mm and were made from folded aluminum foil. The mounts had five 1.5 mm-diameter holes punched into them using 1.5 mm biopsy punches (Miltex) and were distributed across its surface as shown in Fig. 5. The mounts where then covered in carbon tape (except over holes) and mounted to a grounded spinning metal drum that was 62.5 mm in diameter using carbon tape. The drum was spun at 60 RPM and allowed to collect fibers for 60 s. Mounts were then detached from the drum and were imaged with SEM.

Single fiber measurements were acquired using an SEM (Hitachi S4700 SEM, 5 kV, 10 mA, ≈13 mm working distance) and CLSM (Leica SP5 II, Leica Microsystems) with similar settings as during the acquisition of [cell, scaffold] contact data. The single electrospun PLGA microfibers were placed flat on a surface and imaged by SEM at 31.25 nm resolution in X and Y dimensions [1280 pixels × 960 pixels in X and Y] from two viewpoints at 90° and 65° from the flat surface. The two viewpoints allow us to verify that the fibers are cylindrical. After SEM, samples were immersed in PBS and imaged via water immersion CLSM within 2 h of being hydrated (to minimize swelling) since PLGA can swell in buffer. The CLSM z-stacks were acquired at the resolution of 120 nm × 120 nm × 419 nm [2048 pixels × 2048 pixels in X and Y] with approximately 10% spatial area overlap of z-stacks and were manually stitched in a similar method to the SEM 2D images. Figure 5 shows a single fiber sample collector and the SEM and CLSM images acquired along one fiber.

Based on these single fiber measurements, we could validate the segmentation accuracy of fiber scaffolds from CLSM z-stacks against the reference measurements obtained from SEM images. Furthermore, we could use the reference measurements for selecting two of the best models from the eight segmentation models to minimize the time-consuming contact verification effort.

### Methodology

**Fig. 6:**
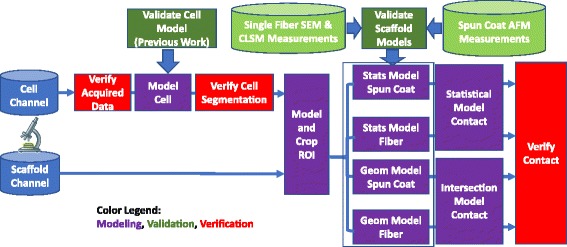
Overview of the measurement methodology for characterization of cell-scaffold contacts. The three components, modeling, validation and verification, are color-coded. The abbreviations “Stats” refers to statistical, “Geom” to geometrical, and “ROI” to region of interest

Following our approach to address the multiple challenges of 3D contact measurements, we designed a methodology as shown in Fig. 6. The validation of a cell model refers to our previous work [7].

**Fig. 7:**
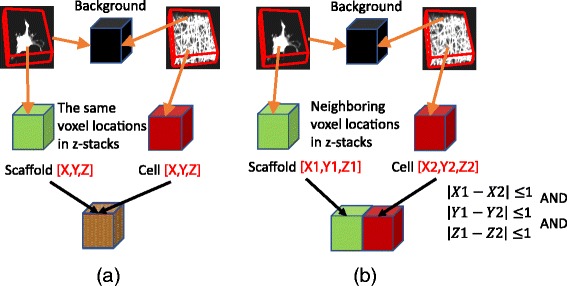
Definition of cell-scaffold contact. Cell and scaffold labels are assigned either (**a**) at the same location or (**b**) at the neighboring locations

In comparison to previous work on co-localization (see Additional file 1), our definition of cell-scaffold contact is aligned with the object-based analysis as opposed to spatial image cross-correlation spectroscopy. While we model objects (cell, scaffold, and background) in two CLSM z-stacks using continuous statistical and geometrical models, the cell-scaffold contact sites are defined based on the spatial proximity of categorical cell and scaffold labels as illustrated Fig. 7. In order to obtain categorical labels, the probability values are adaptively thresholded using maximum entropy criterion [24]:

1 $$ {\mathit{\mathsf{T}}}_{\mathit{\mathsf{opt}}}=\mathit{\mathsf{argmax}}\left\{{\mathit{\mathsf{H}}}_{\mathit{\mathsf{FRG}}}\left(\mathit{\mathsf{T}}\right)+{\mathit{\mathsf{H}}}_{\mathit{\mathsf{BKG}}}\left(\mathit{\mathsf{T}}\right)\right\} $$

where *H*_*FRG*_(*T*) is the entropy of foreground, *H*_*BKG*_(*T*) is the entropy of background, and the optimization is over all values of *T*. The same adaptive thresholding method is used for the geometrical methods after cell masking of the z-stacks processed based on a geometrical model.

### Modeling

Modeling is divided into cell, scaffold, and contact modeling as illustrated in the overview of the methodology in Fig. 6. Cells are segmented using a statistical approach while scaffolds are segmented using multiple statistical and geometrical approaches. Cell-scaffold contacts are obtained based on the law of total probability for statistical models and surface intersection for geometrical models.

#### Cell model for segmentation and ROI model for cropping

We started with cell segmentation by leveraging the previous work [7] and using the permutation-based design of an optimized algorithm selected based on analyses of thousands of cell z-stacks. The algorithm is provided in Additional file 2. All cell segmentation results were visually inspected for quality assurance using the web-based verification system. Out of 414 cell z-stacks, 15 cell z-stacks were manually segmented using ImageJ since the experts rated the results from the automated segmentation as poor or missed. In order to handle large volumetric data, we cropped regions of interest (ROIs) from cell and scaffold z-stacks according to bounding boxes of visually-verified cell segmentation results. Our assumption was that the cell-scaffold contact points occur only in one-voxel neighborhood of the cell surface, and therefore the rest of z-stacks could be discarded. In order to preserve enough voxels around cells, we added 10% margins on each side of x and y boundaries to the cell bounding box enclosing the verified cell segment. For the z boundary, we analyzed the z-axis intensity profile of a scaffold z-stack and selected the frames with high intensity values. The cropping method is described in Additional file 3.

#### Scaffold models

Our modeling approaches to segmenting scaffolds were divided into statistical and geometrical based on the modeling assumptions incorporated by the algorithms. Scaffold z-stacks can be modeled using similar statistical assumptions to the ones used for segmenting cells. However, the scaffold z-stacks typically have smaller amplitude signals than cell z-stacks and hence are more affected by bleed-through and noise. We designed eight specific models as representative samples of a larger body of image processing models. Our goal was to include a model that was optimal in the context of cell-scaffold contact point estimation. Furthermore, the two types of models allowed us to compare segmentation accuracies derived based on general (statistical models) and scaffold-specific (geometrical model) assumptions. These assumptions reflected the amount of prior knowledge embedded into measurement algorithms and the level of effort required to customize models for each type of scaffold. Table 5 provides a short summary of all models. We describe each statistical method in Additional file 5 and provide the algorithmic details in Additional file 6. The geometrical methods are described in Additional file 7 (Algorithm based on planar geometrical model for segmenting spun coat scaffolds) and in Additional file 8 (Algorithms based on cylindrical geometrical models for segmenting fiber scaffolds).

**Table 5:** Summary of statistical and geometrical models for segmenting scaffolds. Note: The geometrical algorithms A6 and A7 with an asterisk are based on modified Frangi vesselness applied to microfiber and medium microfiber scaffolds, and combined with the plane least squares fitting to spun coat scaffolds

Channel treatment	Statistical Models	Geometrical Models of Spun Coat & Fiber Scaffolds
Independent single channel segmentation/labeling	• A1: Single-pixel model	• A6*: Plane & Vesselness (*σ*=1.0)
	• A2: Mixed-pixel spatial model	
		• A7*: Plane & Vesselness (*σ*=1.5)
	• A4: Additive noise model	
	• A5: Markov Random Field model	
Joint two channel segmentation/labeling	• A3: Mixed-pixel channel model (scaffold stain bleed-through or cell stain bleed-through)	• A8: Ad-Hoc Thresholding + Filtering

#### Cell-scaffold contact model

For the statistical models, the contact probability is computed according to the law of total probability by:

2 $$ \mathit{\mathsf{P}}\left(\mathit{\mathsf{Contact}}\right)=\mathit{\mathsf{P}}\left(\mathit{\mathsf{Contact}}|\mathit{\mathsf{Cell}}\right)\;\mathit{\mathsf{P}}\left(\mathit{\mathsf{Cell}}\right)+\mathit{\mathsf{P}}\left(\mathit{\mathsf{Contact}}|\mathit{\mathsf{Scaffold}}\right)\;\mathit{\mathsf{P}}\left(\mathit{\mathsf{Scaffold}}\right). $$

**Fig. 8:**
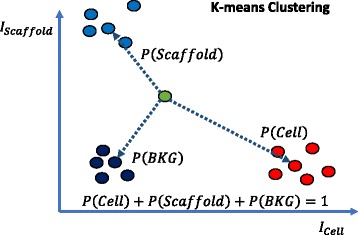
Illustration of probability assignments of cell, scaffold and background (BKG) for a voxel point in the 2D space of intensity values from cell and scaffold channels

**Fig. 9:**
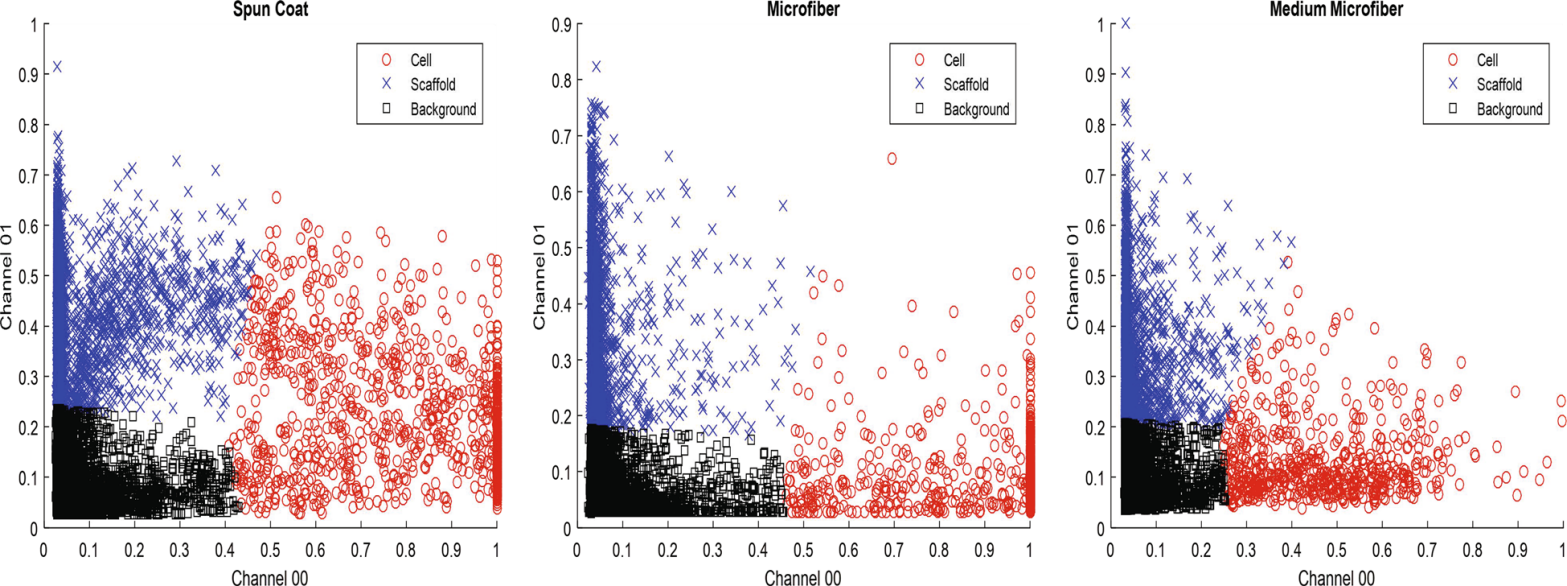
K-means clustering (K = 3) results of voxels in cell and scaffold z-stacks based on their intensities (horizontal axis – intensity of cell or channel 00, vertical axis – intensity of scaffold or channel 01). The three graphs show the distribution of clustering labels for one example from each of the three scaffold types. For the visualization purpose, we randomly sampled 0.1% of the points (27 K points) out of about 27 million points

The aforementioned five statistical models yield two conditional probabilities: *P*(*Contact*| *Cell*) from cell channel and *P*(*Contact*| *Scaffold*) from scaffold channel. In order to estimate the probabilities of *P*(*Cell*) and *P*(*Scaffold*), we use K-means clustering to partition 2D data points formed by intensity values from cell and scaffold channels at each voxel location (i.e., the cell-scaffold intensity scatterplot). Figure 8 illustrates 3 clusters corresponding to cell, scaffold, and background. The probabilities at each voxel point are defined as relative distances to the cluster centroids, constrained by the sum of probabilities equal to 1 (i.e., *P*(*Cell*) + *P*(*Scaffold*) + *P*(*BKG*) = 1). Figure 9 shows examples of cluster assignments of voxel points for each of the scaffold types where the scatterplot points are color-coded as cell (red), scaffold (blue), and background (black) according to the K-means clustering assignment.

For the geometrical models, contact surfaces are the ultimate objective of the measurement. We define the contact model for any geometrical method as the intersection of a binary cell segment with a binary scaffold segment (denoted as geometrical intersection model). Due to the discrete nature of z-stacks, the intersection is defined as a co-occurrence or one voxel adjacency of cell-scaffold binary labels at the same voxel location as illustrated in Fig. 7.

While a plane as a contact surface for spun coat is clearly defined, a contact surface for fibers (MF and MMF) can be defined in multiple ways. A piece-wise linear cylinder can be strictly defined by its skeleton points and a set of radii at those points. It can also be defined in a relaxed sense as a set of voxels obtained by thresholding z-stacks after a vesselness/tubeness filter has been applied. The vesselness filter is based on eigenvalue decomposition of Hessian matrix. This filter computes Hessian at every pixel (voxel) of the input image by convolving the image with second and cross derivatives of the Gaussian function [25]. The sigma parameter (the standard deviation of the Gaussian function) has an impact on the enhanced image appearance. The vesselness filter enhances intensities of tubular structures with radii corresponding to the sigma value. This enhancement is important for selecting a set of tubular voxel candidates in a z-stack by thresholding. Given the uncertainty of contact measurements due to spatial resolution and contact representation (i.e., a cylinder represented with a sequence of spheres at each skeleton point), we opted for a simpler relaxed cylindrical model. To identify the surface points, we computed a 3D gradient for the cell-masked and thresholded scaffold z-stacks and then reported those contact surface points that have non-zero gradient values.

### Validation

#### Validation of geometrical models

The validation of a planar geometrical model for SC scaffold is performed directly by comparing the surface roughness reference measurements from AFM images with the voxel dimensions of each CLSM z-stack. If the surface roughness is smaller than voxel dimensions, then the planar model is suitable. Similarly, the validation of a cylindrical geometrical model for fiber scaffolds (MF and MMF) is achieved by comparing diameters of a single fiber from multi-view 2D SEM images.

#### Assessing accuracy of fiber scaffold segmentation

Given five statistical models and three geometrical models, we compare their accuracy and select one model for each category to minimize the contact verification effort. The accuracy assessment is achieved by measuring the accuracy of algorithms on the single fiber data acquired in SEM and CLSM imaging modalities (see section "Algorithmic model validation measurements". The validation is performed by extracting radius measurements along a single fiber (multiple fields of view) and comparing the radius histogram obtained from the eight algorithms applied to CLSM z-stacks to the radius histogram obtained from 2D SEM images.

The validation methodology consists of the following steps:

1. acquire multiple spatially overlapping fields of view (FOVs) from a sample with single fibers in SEM and fluorescent modalities described in section "Algorithmic model validation measurements".
2. process 2D SEM images to extract radius measurements,
3. process 3D CLSM z-stacks to extract radius measurements,
4. rank-order the designed algorithms applied to the CLSM z-stacks based on the comparison of their radius histograms with the radius histogram derived from the SEM images.

**Fig. 10:**
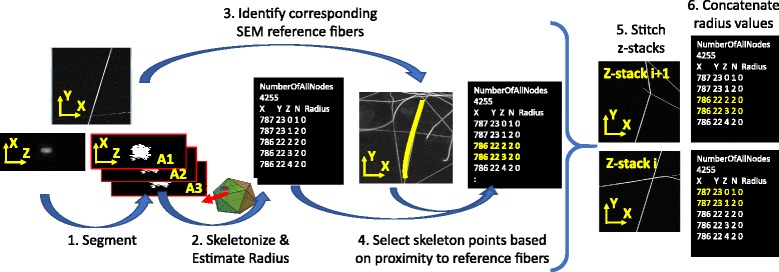
The processing steps applied to CLSM z-stacks of single fiber measurements to estimate radius values

The above processing steps involve stitching multiple FOVs, fiber segmentation, skeletonization of fiber segments, identification of the main reference fiber, and selection of fiber skeleton points that correspond to the main reference fiber. Figure 10 illustrates the sequence of steps to extract radii from CLSM z-stacks (i.e., step 3 of the validation). The entire validation sequence is detailed in Additional file 10.

### Verification of cell segmentation and cell-scaffold contacts

Due to the large volume of [cell, scaffold] image data, we employed automated software-based contact point measurements. As a performance evaluation of the software, an efficient mechanism for visually verifying all contact results was devised since it is very difficult to create ground truth for 3D contact points. The challenges of designing such a verification system include:

1. 3D inspection from multiple view angles,
2. simultaneous presentation of co-registered 3D channels and contacts,
3. access to the verification system from multiple remote locations due to geographically distributed experts, and
4. definition of verification labels to assure consistency of label assignment.

These verification challenges must be resolved under the constraints of minimum verification time and maximum accuracy.

**Fig. 11:**
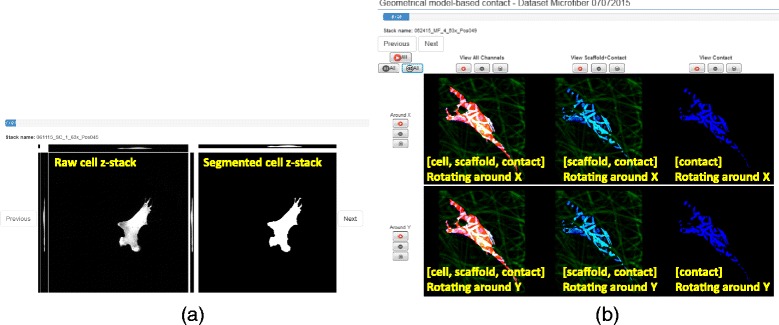
Examples of visual verification in the web system. **a** - cell segmentation (projections of raw and segmented cell z-stacks). **b** - cell-scaffold contact (6 animations showing various combinations of information and rotating around X and Y axes)

To address the first challenge, we designed a web-based verification system for cell segmentation and cell-scaffold contact. For cell segmentation, the multiple view challenge is addressed by presenting side-by-side three orthogonal max projections of raw cell and cell segment z-stacks per cell. The max projections are sufficient to verify the shape accuracy of cell segments because the cell processing steps are designed to report a compact cell shape. For contacts, the same challenge is tackled by creating six web-page embedded movies per [cell, scaffold] pair. However, due to the 3D complexity of contact shapes, max projections are insufficient for contact verification. We opted for creating animations to convey multiple views and to accommodate the time vs. accuracy constraints. Animations are accompanied by controls that allow the movies to play, pause, and rewind, as well as to synchronize any subset of them. Figure 11 displays examples of the web-based verification of cell segmentation and cell-scaffold contacts.

The second challenge of simultaneous presentation of co-registered channels is only relevant to contact verification. It is addressed by forming pseudo-color video frames that contain information about cell, scaffold and their contact. The semantic meaning of [red, green, blue] pseudo-colors is overlaid in yellow text on the videos in Fig. 11. Furthermore, the cell and scaffold channels have different dynamic ranges which affect the rendering. To determine the optimal value for gamma correction, we performed a small user study using a set of z-stacks enhanced by a range of gamma values and presented as movies.^1^ Based on the user study, the gamma value for correcting scaffold intensities was set to 1.4 in all movies presented for contact verification.

The third challenge of accessing the verification system is approached by designing a web solution. The design uses the AngularJS JavaScript library [26] that supports declaring dynamic views in web-applications (transitions between any two data sets for verification). The web solution also leverages the current support of movie formats in HTML5 web technology.

To address the fourth challenge and establish consistent verification of labels across multiple viewers, it is important to define quantitative metrics for all labels. Although the verification labels are assigned subjectively, they are defined as percentages or ratios of voxels that are accurately assigned to a cell or a contact based on a visual inspection. The labels for cell segmentation are created by thresholding the percentage of correctly labeled cell pixels at [90%, 100%] (“good”), [75%, 90%) (“correct”), and [0%, 75%) (“incorrect”), and by recognizing missed cells with a label “missed.” The case of “missed” occurs when multiple cells are in one FOV and the segment of interest is not selected by the algorithm. The labels for contacts are expressed in terms of error ratios with respect to the total volume (statistical model) or surface (geometrical model) of a cell as [0, 1/12] (“excellent”), [1/12, 1/3] (“acceptable”) and [1/3, 1] (“bad”).

## Results

The experimental results are presented in the order of steps that the cell-scaffold contact methodology is executed. The steps are denoted to map to the methodology overview shown in Fig. 6.

### Model and segment cell

The cell segmentation algorithm was executed on all 414 cell channel z-stacks (see Additional file 2 and [7]). The segmentation computation took 84.5 h (24 h for 165 SC cells, 33 h for 135 MF cells, and 27.5 h for 114 MMF cells). The time was benchmarked using a single threaded Java program, Mac OS X, Mac Pro desktop computer (CPU: 3.2 GHz Quad-Core Intel Xeon, RAM: 16 GB 1066 MHz DDR3, and data residing on a network server with 1 Gbit/s bandwidth).

### Verify cell segmentation results

To verify the quality of cell segmentation [7], we deployed a web-based system on a public NIST server at https://isg.nist.gov/CellScaffoldContact/app/index.html. The web-based system contains 414 cells that have been labeled by three cell biologists for this study. We summarized the ratios of the assigned label agreement by any two experts in Table 6.

**Table 6:** Summary of cell segmentation verification in terms of the ratio that two experts assign the same label where the label is from (a) initial label set {good, correct, inaccurate, missed} or (b) combined label set {label1 = {Good or correct}, label 2 = {incorrect or missed}}

Ratio of Matching labels	Expert 2		Expert 3	
	Initial label set	Combined label set	Initial label set	Combined label set
Expert 1	0.86	0.94	0.82	0.95
Expert 2	1	1	0.87	0.94

Following the precision computation in [7] and based on the values in Table 6 the cell segmentation precision per initial label set is (0.86 + 0.82 + 0.87)/3 = 0.85. Similarly, the cell segmentation precision per combined label is (0.94 + 0.95 + 0.94)/3 = 0.943. Out of 414 cell z-stacks, we identified 15 pairs for which all experts assigned the label {incorrect or missed}. Thus, cell segmentation error is estimated as (414–15)/414 = 0.964. These 15 cells were manually segmented using ImageJ/Fiji (plugin crop3D) [27].

### Model and crop region of interest (ROI)

Cell and scaffold z-stacks are cropped according to bounding boxes of visually verified cell segmentation results to reduce the computational time on further processing. The cropping step leads to a significant data size reduction as summarized in Table 7. The cropping also reduces RAM requirements since the dimensions of z-stacks are cut down from 2048 × 2048 pixels in X and Y to (200 to 1906) x (153 to 2045) pixels, and from up to 175 frames in Z to (25 to 114) frames. The number of voxels in one z-stack ranges from 1,827,705 to 188,095,516 voxels.

**Table 7:** Summary of data size reduction after cropping

Dataset	Original Z-Stacks (GB)	Cropped Z-stacks (GB)
SC	304.56	16.23 (5.3% of the original)
MF	396.42	18.46 (4.7%)
MMF	334.84	9.37 (2.8%)
Total	1035.83	44.06 (4.3%)

**Fig. 12:**
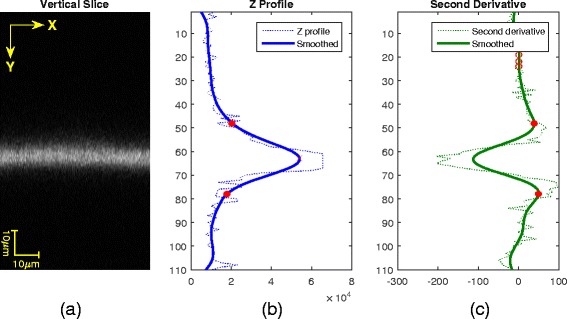
Determination of crop boundary along Z-axis. (**a**) Max projection of scaffold z-stack to [X, Z] plane, (**b**) integrated intensities horizontally along X to obtain a Z-profile, and (**c**) selection of Z frames at the inflection points (red) as the lower and upper boundaries. Note that the aspect ratio of a vertical slice above was changed for better display

Since we assumed that contact points only exist around cells, we derived the cropping box by adding 10% margins to the cell segment dimensions on each of X and Y sides. To derive the Z dimension of a cropping box, we looked at the intensity distributions across frames in scaffold z-stacks. The start and end frames in a z-stack for cropping are determined to be the inflection points in the second derivative of the z-profile closest to the maximum intensity point along z-axis. The z-profile is obtained by computing [X, Z] max projection of scaffold z-stack, integrating intensities horizontally (along the X axis) by taking maximum intensity value at each X, and smoothing the signal by Gaussian filter of size 21 with standard deviation of 5 (empirically determined). The analysis is illustrated in Fig. 12.

### Statistical modeling: Cell-scaffold contact probabilities from 5 methods

**Fig. 13:**
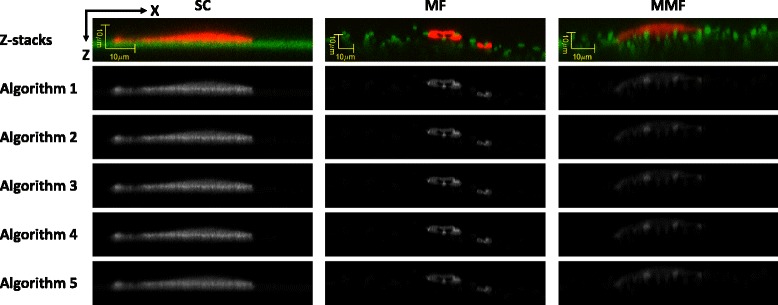
Top row – XZ max projection of pseudo-color coded cell (red) and scaffold (green) intensities for one z-stack from each scaffold type (columns). Five lower rows - [X, Z] max projections of probability values obtained by the five statistical algorithms (rows) where the gray intensity values are mapped between contact probabilities of 0 (black) and 1 (white)

While the algorithms based on geometrical models use implicit shape assumptions, the algorithms based on statistical models use assumptions about intensity models for background. To estimate parameters of a background intensity model, we performed a set of experiments described in Additional file 4 and then derived the average and standard deviation of background from either the first or the last frame of a z-stack (see the algorithms in Additional file 6). Examples of probability results of the five statistical methods are shown in Fig. 13. The figure illustrates that all five algorithms produce visually similar results with a single view, indicating the need for multiple viewing angles for visual verification.

**Fig. 14:**
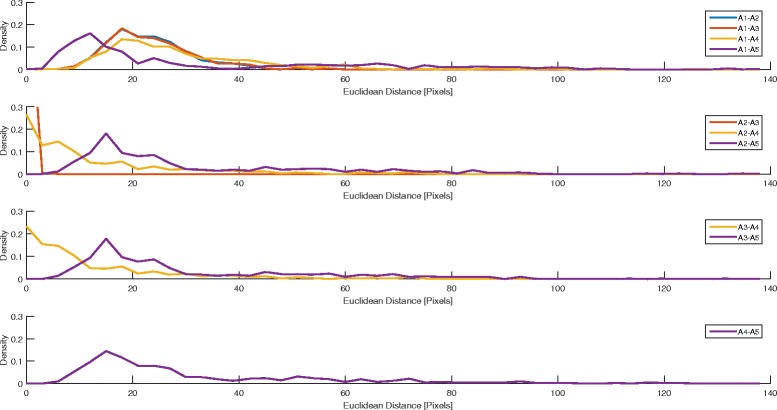
Histogram of Euclidean distances between the probability values generated by any two statistical algorithms (probability density versus Euclidean distance). Alg2 and Alg3 (red line in the second row) exhibit the most similar results

To compare the results quantitatively, we computed Euclidean distance *d*_*ij*_ between contact point probability estimations from algorithms *i* and *j* using the following equation:

3 $$ {\mathit{\mathsf{d}}}_{\mathit{\mathsf{i}\mathsf{j}}}=\sqrt{\frac{\sum_{\mathit{\mathsf{z}}=\mathsf{1}}^{\mathit{\mathsf{Z}}}{\sum}_{\mathit{\mathsf{y}}=\mathsf{1}}^{\mathit{\mathsf{Y}}}{\sum}_{\mathit{\mathsf{x}}=\mathsf{1}}^{\mathit{\mathsf{X}}}{\left({\mathit{\mathsf{p}}}_{\mathit{\mathsf{i}}}\left(\mathit{\mathsf{x}},\mathit{\mathsf{y}},\mathit{\mathsf{z}}\right)-{\mathit{\mathsf{p}}}_{\mathit{\mathsf{j}}}\left(\mathit{\mathsf{x}},\mathit{\mathsf{y}},\mathit{\mathsf{z}}\right)\right)}^{\mathsf{2}}}{\mathit{\mathsf{X}\mathsf{YZ}}}} $$

where *p*_*i*_(*x*, *y*, *z*) is the contact point probability estimated by algorithm *i*. Fig. 14 and Table 8 summarize the Euclidean distances of the results from the five statistical-model based algorithms. The Euclidean distance results correspond to an integral in Table 8 and histogram distribution in Fig. 14 computed from 414 cropped z-stacks (around 11 × 10^11^ voxels) and all pair-wise combinations of algorithms A1 to A5. Based on the integral value for A2-compared-to-A3 (A2-A3) equal to 0.53 in Table 8, we concluded that A2 and A3 algorithms have very similar probability assignments.

**Table 8:** Euclidean distance between the probability of contact point estimations generated by any two statistical algorithms

Euclidean distance [pixels]	A2	A3	A4	A5
A1	24.71	24.86	30.78	33.14
A2	―	**0.53**	13.69	32.70
A3	―	―	13.77	32.60
A4	―	―	―	32.69

The methods were implemented in Matlab 2015a and their computational times are documented in Table 9. The benchmarks were acquired on a desktop computer running Ubuntu 14.04 operating system with Intel Xeon E5–269 2.4 GHz (8 processors), 32 GB of RAM, and all z-stacks stored on an external drive connected via USB3. Note from the second plot in Fig. 14 that the A2-A3 (red) trace is on the x-axis. This allows us to eliminate the A3 statistical algorithm since the accuracy is similar to A2 while its computational time is on average 22.1% higher than the execution time of A2.

**Table 9:** Computational times of the five statistical algorithms for processing all pairs of cells and scaffold z-stacks

Algorithm	Min. Time (s)	Avg. Time (s)	Max. Time (s)
A1 (Single-pixel model)	1.76	21.98	103.31
A2 (Mixed-pixel spatial model)	1.78	24.60	117.85
A3 (Mixed-pixel channel model)	2.09	31.58	143.29
A4 (Additive Gaussian noise model)	2.65	30.82	162.77
A5 (MRF)	3.98	56.24	491.07

### Geometrical modeling: Cell-scaffold contact from 3 methods

Following the plane model and its corresponding algorithmic implementation in Additional file 7, we computed the plane coefficients for the upper and lower surfaces of each spun coat z-stack. To quantify the goodness-of-fit for weighted least squares, we computed Residual Standard Deviation ${STD}_k^{RES}$ per spun coat z-stack and Pooled Standard Deviation *STD* ^*POOLED*^ for all SC scaffolds as follows:

4 $$ {\mathit{\mathsf{STD}}}_{\mathit{\mathsf{k}}}^{\mathit{\mathsf{RES}}}=\sqrt{\frac{\sum \limits_{\mathit{\mathsf{i}}=\mathsf{1}}^{{\mathit{\mathsf{n}}}_{\mathit{\mathsf{k}}}}{\mathit{\mathsf{w}}}_{\mathit{\mathsf{k}\mathsf{i}}}{\mathit{\mathsf{f}}}_{\mathit{\mathsf{k}}}{\left({\mathit{\mathsf{x}}}_{\mathit{\mathsf{i}}},{\mathit{\mathsf{y}}}_{\mathit{\mathsf{i}}},{\mathit{\mathsf{z}}}_{\mathit{\mathsf{i}}}\right)}^{\mathsf{2}}}{\sum \limits_{\mathit{\mathsf{i}}=\mathsf{1}}^{{\mathit{\mathsf{n}}}_{\mathit{\mathsf{k}}}}{\mathit{\mathsf{w}}}_{\mathit{\mathsf{k}\mathsf{i}}}-\overline{{\mathit{\mathsf{w}}}_{\mathit{\mathsf{k}}}}\mathit{\mathsf{p}}}} $$

5 $$ {\mathit{\mathsf{STD}}}^{\mathit{\mathsf{POOLED}}}=\sqrt{\frac{\sum \limits_{\mathit{\mathsf{k}}=\mathsf{1}}^{\mathit{\mathsf{K}}}\left({\mathit{\mathsf{n}}}_{\mathit{\mathsf{k}}}-\mathsf{1}\right)\;{\left({\mathit{\mathsf{STD}}}_{\mathit{\mathsf{k}}}^{\mathit{\mathsf{RES}}}\right)}^{\mathsf{2}}}{\sum \limits_{\mathit{\mathsf{k}}=\mathsf{1}}^{\mathit{\mathsf{K}}}{\mathit{\mathsf{n}}}_{\mathit{\mathsf{k}}}-\mathit{\mathsf{K}}}} $$

where *w*_*ki*_ is the weight at position (*x*_*i*_, *y*_*i*_, *z*_*i*_) in the *k*-th z-stack, $\overline{w_k}$ is the average weight of all voxels in the *k-*th z-stack, *f*_*k*_(*x*_*i*_, *y*_*i*_, *z*_*i*_) = *ax*_*i*_ + *by*_*i*_ + *cz*_*i*_ + *d* is the estimated point in a plane, *p* = 3 is the number of independent parameters in the plane model, *f*_*k*_(*x*_*i*_, *y*_*i*_, *z*_*i*_), *K* = 165 is the number of SC scaffold type z-stacks, and *n*_*k*_ is the number of voxels in the *k-*th z-stack. The minimum and maximum residual standard deviations ${STD}_k^{RES}$ are 54.1 nm and 188.2 nm respectively. The pooled standard deviation *STD* ^*POOLED*^ is 105.1 nm. The distribution of residual standard deviations, as well as alignment of planar surface with the data, are included in Additional file 8.

**Fig. 15:**
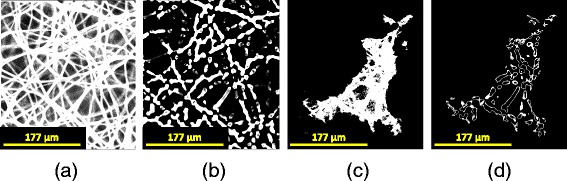
Surface cell-scaffold contact (**d**) derived from the original scaffold z-stack (**a**) after applying modified Frangi’s vessel enhancement filtering (**b**) and verified cell mask (**c**). The results correspond to the method A6 and medium microfiber scaffold type (illustration shows one frame of a z-stack)

Figure 15 shows intermediate results of the geometrical model-based algorithm A6. They include a z-stack after modified Frangi’s vessel enhancement filtering, cell masking, and thresholding and 3D gradient computation. Due to the 3D nature of the contact surfaces and the large number of fibers intersecting the cell segment, it is hard to visually assess the contact quality from a single frame. To facilitate visual inspection of scaffold segmentation and cell-scaffold contacts, we applied post-processing (skeletonization, radius estimation) and represented the fibers as a sequence of spheres extruded along the skeletal points. However, the additional post-processing steps introduce several sources of uncertainties in contact detection and therefore we used the results shown in Fig. 15 for further processing. During the experimentation, we visually compared the performance and parameter choices of the vesselness methods by Frangi [28], by Sato [29] and by Erdt et al. [30] before choosing the Frangi’s method. We have also considered 2D steerable filters [31] and their 3D extensions [32]. While the 3D steerable filters are theoretically related to the vesselness filters, their on-line available implementation requires much more CPU and RAM resources than the vesselness filter implementation (according to the on-line available implementation of [32], minimum RAM must be at least 17 times the original volume size). Based on our visual comparison of the steerable filters and vesselness filters, the steerable filters underperformed vesselness filters in detecting cylindrical fiber surfaces.

### Validation of planar and cylindrical geometrical models

From the AFM measurements described in section "Algorithmic model validation measurements", we computed the average RMS and its standard deviation for spun coat films to be 52.35 nm ±31.76 nm. These statistics are calculated from six spatially distributed spots (RMS: 109, 59, 43.2, 50.6, 39, 13.3). For the voxel resolution of z-stacks as 120 nm × 120 nm × 462 nm, the voxel dimensions are 2 to 8 times larger than the average RMS and its standard deviation. This is supporting our conclusion that the use of a planar geometrical model for spun coat scaffolds is appropriate.

The single fiber SEM measurements from two imaging angles described before allowed comparison of fiber diameters extracted using DiameterJ (plugin to ImageJ/Fiji [33]). The differences in fiber diameters were within 3% error introduced by SEM image processing needed to extract diameters. Thus, the assumption of a cylindrical fiber model is appropriate.

### Assessing fiber scaffold segmentation accuracy using single fibers

We followed the processing workflow shown in Fig. 10. The 2D SEM image analyses are based on ImageJ/Fiji library [27] and DiameterJ (plugin to ImageJ/Fiji [33]) while the 3D CLSM z-stack analyses are based on in-house implementations. The stitching vector is constrained to translation and has been estimated using (a) automated stitching (max projection and pair-wise stitching, max projection and grid stitching, 3D z-stack pair-wise correlation on 4× down-sampled data), (b) semi-automated stitching by defining pairs of corresponding points, and (c) manual stitching using max projection and visual alignment of tiles. The skeletonization is based on 3-D medial axis thinning algorithms [34]. The radius estimation is computed as the smallest eigenvalue of a covariance matrix from all point coordinates selected using an equal angular spacing in 2D or 3D.

**Fig. 16:**
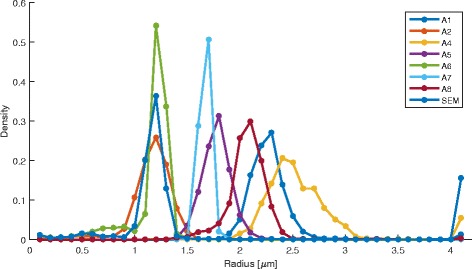
Histograms of single fiber radius measurements extracted from fluorescent CLSM z-stacks using the statistical and geometrical algorithms and from SEM using ImageJ/Fiji plugins

Due to the challenges related to stitching FOVs containing straight lines (i.e., stitching offset uncertainty), we evaluated statistics of radius histograms from two sets of detected fiber skeleton points. The two sets contain skeleton points from either all z-stack FOVs (denoted as ALL) or non-overlapping parts of z-stack FOVs determined based on estimated stage position and approximate stitching vectors (denoted as Internal). Following the validation steps presented in section "Validation" and Additional file 10, the histograms of radii for the set denoted as ALL is shown in Fig. 16 and the comparative summary of histogram statistics is presented in Table 10.

**Table 10:** Summary of radius results extracted from CLSM by eight algorithms and compared to the reference radius measurements from SEM

Fluorescent Input	Radius ± stdev Estimate [um]		Number of points	
Algorithm	ALL: Average ± stdev	Internal: Average ± s tdev	ALL	Internal
A1 (stats: single pixel)	2.2795 ± 0.6151	2.3075 ± 0.7189	32,608	22,128
**A2 (stats: mixed pixel)**	**1.1190 ± 0.3128**	**1.1562 ± 0.3464**	**32,608**	**22,128**
A4 (stats: noise model)	2.6851 ± 1.3196	2.7745 ± 1.5440	32,608	22,128
A5 (stats: MRF)	1.7532 ± 0.3353	1.7648 ± 0.3843	32,608	22,128
**A6 (geom: σ= 1.0)**	**1.1139 ± 0.1960**	**1.0815 ± 0.2112**	**36,249**	**25,709**
A7 (geom: *σ*= 1.5)	1.6251 ± 0.1065	1.6136 ± 0.1175	30,105	20,064
A8 (geom: thresh)	2.0404 ± 0.3567	2.0674 ± 0.4018	32,138	21,691
**SEM Input**	**Average Radius: 1.1242 ± 0.075**		**Number of points: 104,341**	

Based on the single fiber experiments, we concluded that the mixed-pixel statistical model A2 and the vesselness geometrical model A6 (with *σ*= 1.0) applied to fluorescence CLSM z-stacks resulted in the closest average radius estimates to the SEM based average radius. The SEM radius estimate is obtained from 104,341 skeleton points while the CLSM radius estimates come from 20,000 to 36,000 skeleton points. Given the ratio of SEM to CLSM spatial resolutions 0.12/0.0312 = 3.84, the one-to-one match between SEM and CLSM skeleton points would be 104,341/3.84 ≈ 27,000 CLSM points. The standard deviation of the SEM radius is 0.075 while the standard deviation for the method A2 is 0.31 and 0.35, and for the method A6 is 0.20 and 0.21. The ratios of radius standard deviations CLSM/SEM (A2:[4.13, 4.67], A6:[2.67, 2.80]) should theoretically be close to the ratio of spatial resolutions 3.84. The maximum difference between 3.84 and ratio values within the ranges are larger for the method A6 than for the method A2 (3.84–2.67 = 1.17 > 4.67–3.84 = 0.83). This reflects the fact that the A6 model is more constrained (selects only voxels that meet the vesselness model).

With respect to the SEM based estimates, the error of segmenting a single fiber from fluorescent CLSM and estimating its radius using the statistical A2 method is between |1.1242–1.1190|/ 1.1242 = 0.46% and |1.1242–1.1562|/1.1242 = 2.85%. The same error for the geometrical A6 method is |1.1242–1.1139|/1.1242 = 0.92% and |1.1242–1.0815|/ 1.1242 = 3.80%. These errors range between 0.46% to 3.8% of the SEM radius while our visual estimate of SEM radius is about 3%. When the SEM-based errors are compared to the CLSM- based radius standard deviations of the two methods (A2:[0.31, 0.35], A6:[0.2, 0.21]), the errors represent not more than 9.19% and 19% of each method’s one standard deviation respectively (A2: 0.0285/0.31 = 0.0919, A6: 0.0380/0.2 = 0.1900).

### Verification of cell-scaffold contact sites

The web-based verification system described in section "Verification of cell segmentation and cell-scaffold contacts" was populated by six movies per [cell, scaffold] pair, which yields 414 × 6 movies = 2484 movies. This number of movies is generated for each of the two selected contact methods A2 and A6. Each movie is constructed by generating 128 frames of size (640 × 640) pixels, 3 color channels, and presented at 15 frames per second. The movies are compressed from 157.3 MB (640 × 640 × 3 bytes × 128 frames = 157.3 MB) to 2.6 MB video in MP4 H264 codec with visually acceptable blur at 4000 bit rate. The video viewing time is about 9 s. Total movie time is 372.6 min = 6.21 h per method. An on-line help document is available to understand the movie content using pseudo colors, spatial layout of movies, and movie controls.

The movie frame generation is accomplished by data loading using ITK^2^ library with libNifti^3^ loader, creating a window using Qt library and QtCreator^4^ environment, rendering the window content using OpenGL^5^ and then saving frames with OpenCV^6^. The generated frames are aggregated into a movie using ffmpeg library.^7^ Computational benchmarks of the movie generation are summarized in Table 11. The benchmarks were collected on Ubuntu 16.04 64-bit operating system with 49.5GB RAM, 16 processors; Intel® Xeon® CPU E5620@2.4GHz, 2× GF 100GL [Tesla C2050/C2070] NVIDIA card with 6 GB of RAM, and 1× GeoForce GTX 760 NVIDIA card with 2 GB of RAM.

**Table 11:** Computational benchmarks of generating the verification movies. Note: 3 movie creation jobs were running in parallel

Scaffold Type	SC	MF	MMF
Number of [cell, scaffold, contact] triplets	165	135	114
Movie creation time	4 h:40 min	4 h:47 min	2 h:55 min

The visual verification was conducted by three experts over two contact detection methods (Statistical A2 and geometrical A6) and three scaffold types. The labels for each cell-scaffold contact detection span excellent, acceptable and bad. For the statistical model-based method, the following labels were defined:

- Excellent: Visually, error is not exceeding 1/12th of the total volume of cell.
- Acceptable: Visually, combined errors do not exceed ~1/3rd of the total volume of cell.
- Bad: Visually, combined errors exceed ~1/3rd of the total volume of cell.

**Fig. 17:**
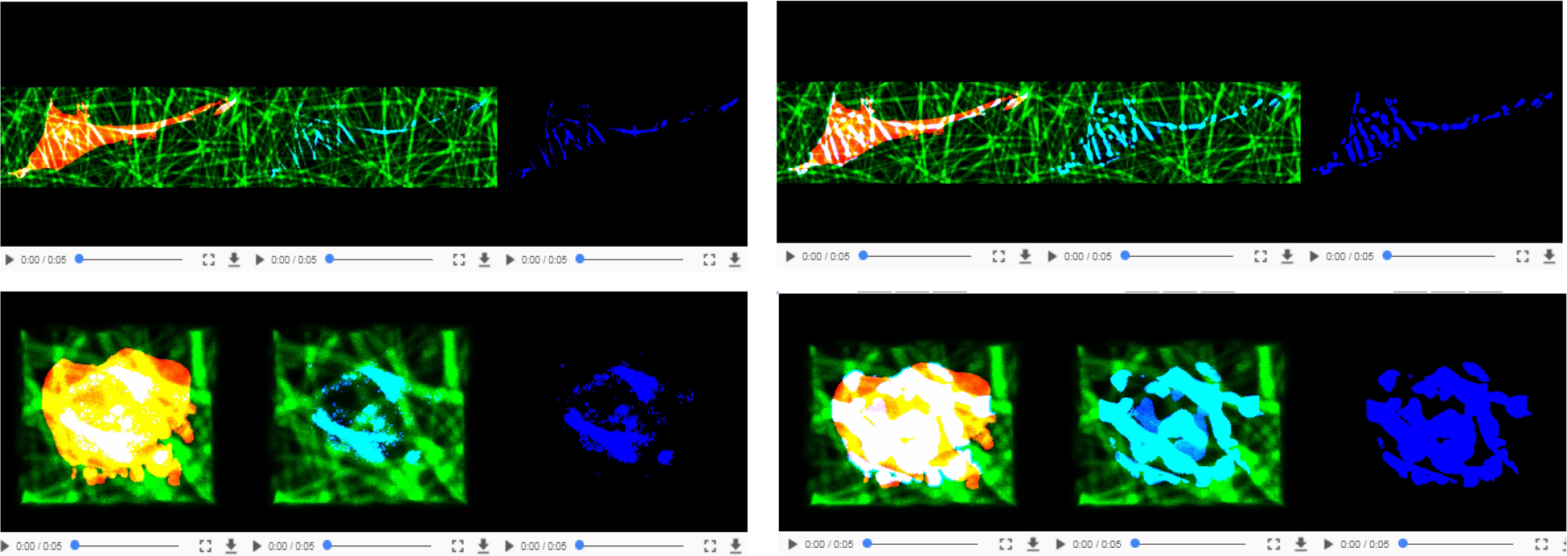
Two examples of cell-scaffold contacts that were labeled by all three experts as excellent (top) and bad (bottom) for both Statistical A2 (left) and Geometrical A6 (right) methods

For the geometrical model-based method, the labels were defined in the same way but the total volume of cells was replaced by the total surface of cell. Figure 17 illustrates two cell-scaffold contact examples that were unanimously labeled by all three experts as excellent (top) and bad (bottom) for both Statistical A2 and Geometrical A6 methods.

**Fig. 18:**
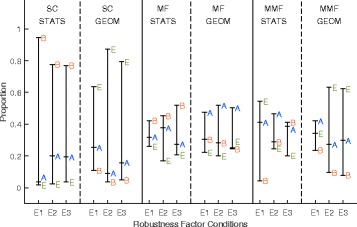
Summary of contact verification for three scaffold types (SC, MF, MMF), two models (Stats A2 and Geometrical A6), and three experts (E1, E2, and E3). The vertical values are proportions (percentages) of excellent, acceptable and bad labels per one of configurations of the three factors (X1: scaffold type, X2: model, X3: expert)

The total time to complete the verification by the three experts was 6 h + 8 h + 6 h = 20 h. The results of visual verification were reported as proportions of the three labels (excellent, acceptable, bad) per model (A2, A6), scaffold type (SC, MF, MMF) and expert (E1, E2, E3) in Fig. 18. The proportional values are most distinguishable for SC, most compressed for MF, and most unpredictable for MMF. For SC scaffold type and excellent rating, geometrical model A6 is clearly better than statistical model A2. For MF scaffold type, the dominant rating is poor for statistical model while it is acceptable for geometrical model. For MMF scaffold type, the ranking of proportion values varies across experts.

Overall accuracy of contact measurement was defined as the ratio of the contacts labeled by at least one of the experts as excellent or acceptable over the total number of [cell, scaffold] pairs. According to this definition, accuracy of the statistical method A2 is (414–155)/414 = 0.626 and accuracy of the geometrical method A6 is (414–27)/414 = 0.935. Precision of contact measurements was derived as an average of the probabilities that two experts agreed on a label. These pair-wise ratios of label agreement are summarized in Table 12. From the results in Table 12, the contact precision for the statistical method (A2) is (0.74 + 0.75 + 0.81)/3 = 0.767 and for the geometrical method (A6) is (0.86 + 0.86 + 0.91)/3 = 0.876.

**Table 12:** Summary of cell-scaffold contact verification in terms of the ratio that two experts assign matching labels from the sets {excellent, acceptable} or {bad}

Ratios of label agreement	Expert 2		Expert 3	
Method	Stat (A2)	Geom (A6)	Stat (A2)	Geom (A6)
Expert 1	0.74	0.86	0.75	0.86
Expert 2	1	1	0.81	0.91

## Discussion

### Quantitative discussion

#### Verification-based accuracy and precision of cell-scaffold contact methods

Based on section "Verify cell segmentation results", we assessed the accuracy of the cell segmentation method based on visual verification of three experts to be 0.964 with precision 0.943 for the two groups of labels {accurate, good} and {inaccurate, missed}. Based on similar assessment of cell-scaffold contacts of labels {excellent, acceptable} and {bad} in section "Verification of cell-scaffold contact sites", the accuracy of a statistical method A2 is 0.626 with 0.767 precision and the accuracy of a geometrical method A6 is 0.935 with 0.876 precision. By comparing the accuracies of cell segments and cell-scaffold contact sites based on visual verifications, the cell segmentation algorithm is more accurate and more precise than the cell-scaffold contact algorithms. These differences present the tradeoffs between the reliability and potential prediction power of cell shape versus cell-scaffold contact shape according to Fig. 1.

#### Validation, model fitting- and verification-based accuracy of planar model for spun coat scaffolds

Based on planar model validation in section "Validation of planar and cylindrical geometrical models", the AFM-derived surface roughness of 52.35 nm ±31.76 nm was 2 to 8 times smaller than the CLSM resolution which supported the use of a planar model. Based on model fitting-based accuracy in section "Geometrical modeling: Cell-scaffold contact from 3 methods", the planar fit of spun coat in measured CLSM z-stacks had the pooled standard deviation *STD* ^*POOLED*^ of 105.1 nm which is smaller than any of the three voxel dimensions (120 nm × 120 nm × 462 nm). The visual verification of SC scaffold type confirmed the low value of pooled standard deviation since the three experts reported only 18, 6, and 8 contacts as “bad” respectively out of 165 pairs which corresponds to 10.91%, 3.64%, and 4.85% of the number of SC scaffolds.

#### Validation-based accuracy of relaxed cylindrical geometrical model for fiber scaffolds

For MF scaffolds, the fiber radius fit was evaluated using the single fiber experiments in section "Assessing fiber scaffold segmentation accuracy using single fibers" and the errors ranged between 0.46% to 3.8% of the SEM radius. These error values were comparable to the 3% radius error from single fiber SEM images based on our visual inspection. The fiber radius in a single fiber experiments was 1.1242 μm ±0.075 μm and can be related to the results of visual verification for MF scaffold (radius ≈ 1.3 μm). The visual verification of MF scaffold type yielded 41, 38, and 33 contacts labeled as “bad” out of 135 pairs (30.37%, 28.15%, and 24.44%). By comparing the algorithmic errors observed from single fiber experiment of fiber radii and from visual verification of contacts, we could conclude that the cell-MF scaffold contact has about 13× worse error than a single fiber radius error (contact: 30.37%, 28.15%, and 24.44% errors per expert versus radius: 0.46% to 3.8% errors per point selection; average contact error/average radius error = 27.65/2.13 ≈ 13). The magnitude of this ratio illustrates the complexity of cell-MF scaffold contact versus single fiber radius measurements and the challenges associated with multiple touching fibers and channel bleed-through. Interestingly, the visual verification of MMF scaffolds (radius ≈ 0.55 μm) led to 27, 11, and 9 contacts labeled as “bad”’ respectively out of 114 pairs (23.68%, 9.65%, and 7.89%) which were smaller errors than those for the MF scaffolds.

#### Computational and human labor costs

As with many “big data” experiments, the computational and human labor costs are not insignificant. To execute all computational steps of the methodology, it took approximately (a) 84.5 h to segment cells and to generate max projections for cell visual verification (data on network drive), (b) 13.33 h to crop data (all z-stacks stored on an external drive connected via USB3), (c) 19 h to run all five statistical methods (A1-A5) on the 414 z-stacks of [cell, scaffold] pairs and obtain probabilities of contacts, (d) 3.45 h to convert statistical probabilities to binary contacts and 17.25 h to convert vesselness filtered values to binary contacts (data on local drive), (e) approximately 3.49 h to run the three geometrical methods (A6, A7, A8) on all 414 pairs (data on local drive), and (f) 12.37 h to generate movies. The total computational time was approximately 153.39 h. The computational times have been collected on five computers and include some input/output overhead in order to accommodate heterogeneous platforms of major contributors. We also approximated the total time spent by the three experts on verifying the cell segmentation was around 4 h + 4 h + 4 h = 12 h and on verifying the cell-scaffold contact sites around 6 h + 8 h + 6 h = 20 h.

### Qualitative discussion

#### Modeling tradeoffs

The cell-scaffold contact methodology consists of modeling, validation and verification with several tradeoffs. The first tradeoff is related to choosing a model: general statistical model versus custom geometrical model. In other words, geometrical models can be specific to each scaffold type (e.g., a planar model for SC and a tube model for MF) while statistical models are more general. Thus, statistical methods can be re-used for other experimental scaffolds while geometrical methods would have to be developed for each type of scaffold.

Another tradeoff is between the labor/computational complexity and the number of plausible contact models included in the search space of models. The word “plausible” should be interpreted with caution because a priori assumptions about plausible models are injected into an algorithm. This is the reason why in order to avoid biases, geometrical models with stronger assumptions about the scaffolds than the statistical models are validated by physics-based orthogonal measurements rather than just by visual verification in our study.

#### Physics-based validation and visual verification tradeoffs

We obtained measurement accuracies based on visual verification and validation using physics-based orthogonal measurements. This poses a tradeoff between the value and cost of the two approaches. The value of visual verification lies in delivering confidence in accuracy measurements at the cost of manual labor. The visual inspection also allows for identifying errors in algorithms and discovering new phenomena. The drawback is that it is a qualitative assessment at a coarse level and that there are differential limitations in visualization quality based on user display. The value of validation using orthogonal measurements lies in removing human bias at the cost of smaller confidence in accuracy measurements because of different measurement conditions. The advantage of validation is in establishing quantitative assessment at a fine (voxel) level.

There is an option of establishing accuracy and robustness by using data-driven simulations. In our case, simulations started with segmentation of existing [cell, scaffold] pair and extracted skeletons and radii of fibers, followed by model-based simulations of cross-channel bleeding, optical distortions and Gaussian noise. However, we are not reporting simulations because they require validating all simulation models, estimating their parameters, and comparing simulations against reference cases to show that the simulations are accurate.

#### Quality control considerations

The quality of cell-scaffold contact measurements depends highly on the quality of data and models. There is a tradeoff between doing quality control after acquisition and after obtaining a contact measurement. In our study, we eliminated 297 z-stacks of [cell, scaffold] pairs from the 711 automatically acquired z-stacks which lowered the computational and verification efforts. The elimination of cells out-of-focus and touching cells took approximately 2 h using CLSM software (Leica LAS AF) for browsing acquired z-stacks. If we did not eliminate the 297 pairs then the total computational time and visual verification time would increase by a factor of 711/414 = 1.72.

Based on our observations, the most detrimental effect on contact measurements comes from channel bleed-through. In the case of bleed-through, we are unable to extract reliable contact measurements as opposed to other cases when the effects can be corrected manually (e.g., cell segmentation of touching cells) or by algorithmic design (e.g., cells extending outside of a FOV).

#### Complexity and heterogeneity considerations

Finally, the complexity of cell-scaffold contact measurements from a TB-sized collection of z-stacks must be addressed by a team with diverse expertise. The diversity leads to a chain of heterogeneous contributions to the final contact measurement in terms of software languages, operating systems, and hardware platforms on which the measurement is performed. Thus, multiple verification milestones become critical to address the complexity and data scale of contact measurement, as well as to eliminate sources of computational errors.

## Conclusions

The described object-based contact measurement methodology enabled (a) optimized cell-scaffold contact representations that incorporate a range of statistical and geometrical models, (b) validated 3D contacts using reference measurements, and (c) visual verification and efficient contact measurement of 414 cell-scaffold interactions with two analysis methods over three types of scaffolds, totaling about 1 TB of data. The key contributions come from (1) the contact modeling and the validation methodology, (2) the large scale of contact measurements with 100% visual verification, and (3) the web mechanism for disseminating and reviewing contact measurements from a TB-sized collection of z-stacks.

In the near future, the resulting well-characterized cell-scaffold contact measurements will be used to extract and classify shape dimensionality, while the methodology and computational parts can be re-used for other co-localization studies. We also plan to compare the accuracy and time needed for contact verification with approaches that utilize the state-of-the-art National Institute for Standards and Technology virtual reality metrology facility.

## Additional files

Additional file 1: Detailed description of related work. (DOCX 59 kb)

Additional file 2: Cell segmentation algorithm. (DOCX 25 kb)

Additional file 3: Model for cropping contact regions of interests. (DOCX 17 kb)

Additional file 4: Statistical model of background. (DOCX 90 kb)

Additional file 5: Statistical models for segmenting all scaffold types. (DOCX 192 kb)

Additional file 6: Algorithms based on statistical models for segmenting all scaffold types. (DOCX 48 kb)

Additional file 7: Algorithm based on planar geometrical model for segmenting spun coat scaffolds. (DOCX 36 kb)

Additional file 8: Algorithms based on cylindrical geometrical models for segmenting fiber scaffolds. (DOCX 25 kb)

Additional file 9: Evaluation of goodness-of-fit for planar model used for modeling spun coat scaffolds. (DOCX 796 kb)

Additional file 10: Validation steps based on 2D SEM and 3D CLSM data of Single Fibers. (DOCX 34 kb)

## Abbreviations

AFM: atomic force microscopy
CLSM: confocal laser scanning microscopy
CPU: central processing unit
FOV: field of view
hBMSCs: human bone marrow stromal cells
ICCS: image cross-correlation spectroscopy
MF: Large Microfibers
MMF: Medium Microfibers
PLGA: poly(lactic-co-glycolic acid)
RAM: random-access memory
SC: Spun Coat
SEM: scanning electron microscopy

## Footnotes

1
https://isg.nist.gov/CellScaffoldContact/app/pages/docs/gammaCorrection.html

2
https://itk.org/ITK/resources/software.html

3
http://niftilib.sourceforge.net/

4
https://www.qt.io/download/

5
https://www.opengl.org

6
https://opencv.org/

7
https://ffmpeg.org/
