# Supplementary material for: Modeling, validation and verification of three-dimensional cell-scaffold contacts from terabyte-sized images
Source: BMC Bioinformatics. 2017 Nov 28;18:526. doi: 10.1186/s12859-017-1928-x (PMC5706418; doi:10.1186/s12859-017-1928-x)
Supplement: Supplementary file 1 — Detailed description of related work. (DOCX 59 kb) [file 12859_2017_1928_MOESM1_ESM.docx]

Additional file 1: Detailed description of related work

**Co-localization:** Our work on modeling can be related to the published methods that focus on the problem of measuring the co-localization of objects in dual-color fluorescent confocal images and to 3D segmentation [1]. The co-localization problem has been investigated since a successive detection of two fluorochromes in the same specimen became possible using a dual-color confocal microscope in 1990 [2]. In the past co-localization studies, the objective has been to estimate the frequency of coincidental objects (i.e., the degree of co-localization). The most frequent reported approaches are based on the computation of Pearson’s correlation coefficient [2], Spearman’s rank coefficient outperforming the Pearson’s coefficient [3], overlap coefficient [1], and blob co-localization as a measure of how groups of associated voxels relate to one another [4]. These approaches are denoted as spatial image cross-correlation spectroscopy (ICCS) [5] but cannot be used when the shape of cell-scaffold contact is of interest. The reason lies in the fact that the co-localization coefficients and metrics do not capture the spatial information of contacts. Instead, one has to focus on co-localization approaches using object-based analysis [1] that require object segmentation and avoid lengthy manual identification of segments.

**Foreground modeling (statistical and geometrical models):** The models for automated segmentation are based on a two-dimensional histogram formed as a scatterplot of two channel intensities. For example, in the work of [6], threshold values for segmenting foreground are derived by performing a statistical significance test. Using this test, one shows that a correlation between two measured channels in the 2D histogram is significantly greater than a correlation between two channels with randomly generated intensities. To overcome the assumption about uncorrelated pixel’s intensity with its neighboring pixels, the authors divide images into independent blocks and then randomly scramble blocks instead of individual pixels to generate the baseline (i.e., two channels with randomly generated intensities). Our modeling approach is related to the work of [6] in a sense that it uses a scatterplot of two channel intensities. It is different by considering multiple statistical models, and then selecting the models that deliver the highest accuracy with respect to orthogonal measurements. Similar to [6], our work is also driven by full automation of contact detection due to the large size of z-stack collection that is needed to report statistically significant results for any further hypothesis. In terms of geometrical models suitable to fiber scaffold segmentation, we have tried many software packages including IvanTK<http://hdl.handle.net/10380/3431>, NeuronJ^[[1]](#footnote-1)^, Simple Neurite Tracer<http://imagej.net/Simple_Neurite_Tracer>, Vaa3D^[[2]](#footnote-2)^, Vascular Modelling Toolkit (VMTK)^[[3]](#footnote-3)^. However, these software packages have been designed for vascular or brain structures and hence do not perform well on fiber scaffolds. Some of them work only in 2D and some require manual identification of starting and ending points which is a tremendous burden for analyzing TB-sized collections of z-stacks.

**Validation of 3D segmentation:** The validation part of our work can also be related to the past efforts on validating three-dimensional (3D) segmentation methods of z-stacks from confocal microscopy [7–12]. Evaluations of automated segmentation have to include accuracy (validity), precision (reliability, repeatability), and efficiency (viability) [13],[14]. In order to evaluate segmentation accuracy, one needs reference measurements. Given the volume and complexity of 3D cell-scaffold contacts, creating references by manual labeling is very time consuming and subjective. It also involves design of special tools and visualization. Another option is to collect reference measurements using orthogonal methods. Researchers have used X-ray micro-computed tomography (μCT) and demonstrated the benefits and limitations of μCT for analysis of cell adhesion and proliferation in polymer scaffolds [15]. The μCT analyses applied to polycaprolactone scaffolds were also compared to reference measurements acquired by SEM and CLSM in [16]. For reference measurements of the sub-micrometer cellular structures, custom microenvironmental selective plane illumination microscopy (meSPIM) has been reported in [17]. In our work, SEM has been used to generate orthogonal reference measurements since it delivers about four times higher spatial resolution than CLSM. In comparison to the past work applied to polymer scaffolds, our segmentation evaluation of Poly lactic-co-glycolic acid (PLGA) microfiber scaffolds was established based on multi-view 2D SEM imaging of a single fiber since the fiber has a circular cross-section.

**Verification of complex 3D contacts:** To our best knowledge, there are no publications in the biological domain where the verification has been conducted over TB-sized z-stack collections and published results could be visually verified by all scientists. Due to many sources of uncertainties in 3D cell-scaffold contact measurements ranging from cell preparation, scaffold preparation [18–21], CLSM imaging, and co-localization analyses, there is a significant value in being able to visually inspect the automatically obtained contacts.

**References**

1. Bolte S, Cordelieres FP. A guided tour into subcellular colocalisation analysis in light microscopy. J. Microsc. 2006;224:13–232.

2. Manders BMM, Verbeek FJ, Aten JA. Measurement of co-localization of objects in dual-colour confocal images. J. Microsc. 1993;169:375–82.

3. Adler J, Pagakis SN, Parmryd I. Replicate-based noise corrected correlation for accurate measurements of colocalization. J. Microsc. 2008;230:121–133.

4. Fletcher PA, Scriven DRL, Schulson MN, Moore EDW. Multi-image colocalization and its statistical significance. Biophys. J. [Internet]. Biophysical Society; 2010;99:1996–2005. Available from: http://dx.doi.org/10.1016/j.bpj.2010.07.006

5. Comeau JWD, Costantino S, Wiseman PW. A guide to accurate fluorescence microscopy colocalization measurements. Biophys. J. [Internet]. Elsevier; 2006;91:4611–22. Available from: http://www.ncbi.nlm.nih.gov/pubmed/17012312%5Cnhttp://www.pubmedcentral.nih.gov/articlerender.fcgi?artid=PMC1779921

6. Costes S V, Daelemans D, Cho EH, Dobbin Z, Pavlakis G, Lockett S. Automatic and quantitative measurement of protein-protein colocalization in live cells. Biophys. J. [Internet]. Elsevier; 2004;86:3993–4003. Available from: http://www.ncbi.nlm.nih.gov/pubmed/15189895

7. Indhumathi C, Cai YY, Guan YQ, Opas M. An automatic segmentation algorithm for 3D cell cluster splitting using volumetric confocal images. J. Microsc. [Internet]. 2011 [cited 2014 Sep 15];243:60–76. Available from: http://www.ncbi.nlm.nih.gov/pubmed/21288236

8. Chen J, Kim O V., Litvinov RI, Weisel JW, Alber MS, Chen DZ. An Automated Approach for Fibrin Network Segmentation and Structure Identification in 3D Confocal Microscopy Images. 2014 IEEE 27th Int. Symp. Comput. Med. Syst. [Internet]. 2014 [cited 2014 Sep 15]. p. 173–8. Available from: http://ieeexplore.ieee.org/lpdocs/epic03/wrapper.htm?arnumber=6881871

9. McCullough DP, Gudla PR, Harris BS, Collins J a, Meaburn KJ, Nakaya M a, et al. Segmentation of whole cells and cell nuclei from 3-D optical microscope images using dynamic programming. IEEE Trans. Med. Imaging [Internet]. 2008;27:723–34. Available from: http://www.pubmedcentral.nih.gov/articlerender.fcgi?artid=2730109&tool=pmcentrez&rendertype=abstract

10. Lin G, Adiga U, Olson K, Guzowski JF, Barnes C a, Roysam B. A hybrid 3D watershed algorithm incorporating gradient cues and object models for automatic segmentation of nuclei in confocal image stacks. Cytometry. A [Internet]. 2003 [cited 2014 Sep 15];56:23–36. Available from: http://www.ncbi.nlm.nih.gov/pubmed/14566936

11. Herberich G, Windoffer R, Leube R, Aach T. 3D segmentation of keratin intermediate filaments in confocal laser scanning microscopy. Annu. Int. Conf. IEEE Eng. Med. Biol. Soc. [Internet]. Boston, MA; 2011. p. 7751–4. Available from: http://www.ncbi.nlm.nih.gov/pubmed/22256135

12. Bajcsy P, Chalfoun ACJ, Halter M, Juba D, Kociolek M, Majurski M, et al. Survey Statistics of Automated Segmentations Applied to Optical Imaging of Mammalian Cells. BMC Bioinformatics. 2015;16:1–28.

13. Fenster A, Chiu B. Evaluation of Segmentation algorithms for Medical Imaging. Annu. Int. Conf. IEEE Eng. Med. Biol. Soc. IEEE Eng. Med. Biol. Soc. [Internet]. 2005. p. 7186–9. Available from: http://www.ncbi.nlm.nih.gov/pubmed/17281935

14. Udupa JK, LeBlanc VR, Zhuge Y, Imielinska C, Schmidt H, Currie LM, et al. A framework for evaluating image segmentation algorithms. Comput. Med. Imaging Graph. [Internet]. 2006 [cited 2014 Sep 2];30:75–87. Available from: http://linkinghub.elsevier.com/retrieve/pii/S089561110500114X

15. Dorsey SM, Lin-Gibson S, Simon CG. X-ray microcomputed tomography for the measurement of cell adhesionand proliferation in polymer scaffolds. Biomaterials [Internet]. Elsevier Ltd; 2009;30:2967–74. Available from: http://dx.doi.org/10.1016/j.biomaterials.2009.02.032

16. Parrilli A, Pagani S, Maltarello MC, Santi S, Salerno A, Netti PA, et al. Three-dimensional cellular distribution in polymeric scaffolds for bone regeneration: A microCT analysis compared to SEM, CLSM and DNA content. J. Microsc. 2014;255:20–9.

17. Welf ES, Driscoll MK, Dean KM, Schäfer C, Chu J, Davidson MW, et al. Quantitative Multiscale Cell Imaging in Controlled 3D Microenvironments. Dev. Cell. 2016;36:462–75.

18. Yarin AL, Koombhongse S, Reneker DH. Bending instability in electrospinning of nanofibers. J. Appl. Phys. 2001;89:3018–26.

19. Nain AS, Sitti M, Jacobson A, Kowalewski T, Amon C. Dry spinning based spinneret based tunable engineered parameters (STEP) technique for controlled and aligned deposition of polymeric nanofibers. Macromol. Rapid Commun. 2009;30:1406–12.

20. Lauricella M, Pontrelli G, Coluzza I, Pisignano D, Succi S. JETSPIN: A specific-purpose open-source software for simulations of nanofiber electrospinning. Comput. Phys. Commun. [Internet]. Elsevier B.V.; 2015;197:227–38. Available from: http://dx.doi.org/10.1016/j.cpc.2015.08.013

21. Chew S, Wen Y, Dzenis Y, Leong K. The Role of Electrospinning in the Emerging Field of Nanomedicine. Curr. Pharm. Des. [Internet]. 2006;12:4751–70. Available from: http://www.pubmedcentral.nih.gov/articlerender.fcgi?artid=2396225&tool=pmcentrez&rendertype=abstract%5Cnhttp://www.eurekaselect.com/openurl/content.php?genre=article&issn=1381-6128&volume=12&issue=36&spage=4751

1. http://www.imagescience.org/meijering/software/neuronj/ [↑](#footnote-ref-1)
2. http://home.penglab.com/proj/vaa3d/home/index.html [↑](#footnote-ref-2)
3. http://www.vmtk.org/ [↑](#footnote-ref-3)
