# Supplementary material for: Modeling, validation and verification of three-dimensional cell-scaffold contacts from terabyte-sized images
Source: BMC Bioinformatics. 2017 Nov 28;18:526. doi: 10.1186/s12859-017-1928-x (PMC5706418; doi:10.1186/s12859-017-1928-x)
Supplement: Supplementary file 2 — Cell segmentation algorithm. (DOCX 25 kb) [file 12859_2017_1928_MOESM2_ESM.docx]

Additional file 2: Cell segmentation algorithm

1. Smoothing: morphological erosion + dilation in XY directions (3x3x3).
2. Minimum error threshold optimization from 1 to 65 535, step 1 according to [1].
3. Remove dark frames at the beginning and at the end of the stack.
   - Threshold and erode frames, discard if number of foreground pixels < 500.
4. Find and apply adaptive threshold according to the minimum error criterion described in [2].
5. Remove objects touching the edges.
6. Find largest object and remove all the other ones.

Extra step: generate orthogonal projections of raw and segmented volume to generate side-by-side images for web-based verification.

**References**

1. Bajcsy P, Simon M, Florczyk S, Simon C, Juba D, Brady M. A Method for the Evaluation of Thousands of Automated 3D Stem Cell Segmentations. J. Microsc. [Internet]. 2015;260:363–76. Available from: http://www.ncbi.nlm.nih.gov/pubmed/26268699

2. Sezgin M, Sankur B. Survey over image thresholding techniques and quantitative performance evaluation. J. Electron. Imaging [Internet]. 2004 [cited 2017 Oct 2];13:146–65. Available from: http://pequan.lip6.fr/~bereziat/pima/2012/seuillage/sezgin04.pdf
