# Supplementary material for: Modeling, validation and verification of three-dimensional cell-scaffold contacts from terabyte-sized images
Source: BMC Bioinformatics. 2017 Nov 28;18:526. doi: 10.1186/s12859-017-1928-x (PMC5706418; doi:10.1186/s12859-017-1928-x)
Supplement: Supplementary file 3 — Model for cropping contact regions of interests. (DOCX 17 kb) [file 12859_2017_1928_MOESM3_ESM.docx]

Additional file 3: Model for cropping contact regions of interests

Given bounding boxes for visually verified cell segments:

1. Add 10 % margins on each side of the cell segment bounding box in the X and Y dimensions.
2. Compute the Z-profile of a scaffold z-stack.
3. Apply Gaussian smoothing filter with a standard deviation of 5 and a window size of 21 pixels. Note that the parameters were determined empirically to include points within two standard deviations into the window and minimize the number of spurious peaks in the second derivative.
4. Calculate the second derivative of the Z-profile.

Determine lower and upper bounds along Z-axis based on the inflection points of the Z-profile.
