# Supplementary material for: Modeling, validation and verification of three-dimensional cell-scaffold contacts from terabyte-sized images
Source: BMC Bioinformatics. 2017 Nov 28;18:526. doi: 10.1186/s12859-017-1928-x (PMC5706418; doi:10.1186/s12859-017-1928-x)
Supplement: Supplementary file 4 — Statistical model of background. (DOCX 90 kb) [file 12859_2017_1928_MOESM4_ESM.docx]

Additional file 4: Statistical model of background

The algorithms based on statistical models use assumptions about intensity models for background. The background model for cell and scaffold channels might depend on the sample being “in” or “out” and the laser being “on” or “off”. To quantify the dependencies, we took images of “sample out” with “laser off’ and compared to images of “sample out” with “laser on”. We observed no difference in intensity histograms at the cell wavelength and a minor offset to larger intensities (≈15 intensity units) for scaffold wavelengths in the case of “laser on”. We concluded that the background histogram intensities do not depend on the laser “on” or “off”. Next, we analyzed images with “sample in” and “laser on”. Histogram analyses in *Figure 1* show that the background and foreground distributions are not normal and the intensity values are larger than for the case “sample out”. Thus, to estimate parameters of a background intensity model, we preferred to use the measured z-stacks and identified either the first or the last frame of a z-stack as the frame containing only background intensities.


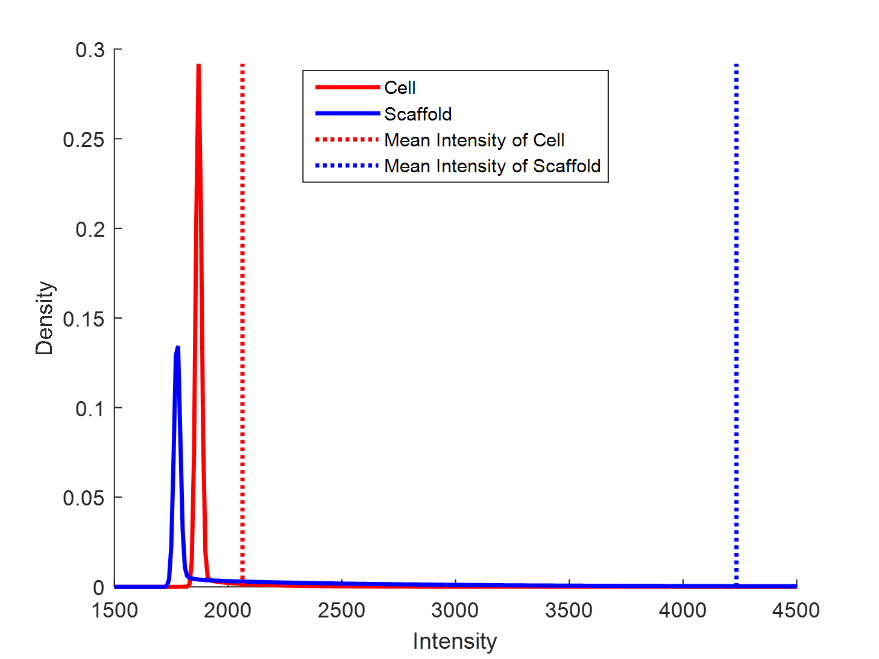


*Figure 1: Histogram analyses to determine the background statistics*
