# Supplementary material for: Modeling, validation and verification of three-dimensional cell-scaffold contacts from terabyte-sized images
Source: BMC Bioinformatics. 2017 Nov 28;18:526. doi: 10.1186/s12859-017-1928-x (PMC5706418; doi:10.1186/s12859-017-1928-x)
Supplement: Supplementary file 5 — Statistical models for segmenting all scaffold types. (DOCX 192 kb) [file 12859_2017_1928_MOESM5_ESM.docx]

Additional file 5: Statistical models for segmenting all scaffold types

**Statistical models:**

Let us denote a pixel location as X = [row, column] and measured intensity at X as $I_{Measured}\left( X \right)$. The contribution from pure background is $I_{BKG}$ and the contribution from pure signal is $I_{FRG}$. The contributions are weighted by coefficients $w_{i}.$

A1: Single-pixel model

- Model: pixels have independent intensities. The probability of a pixel labeled as scaffold is proportional to its measured intensity $\frac{I_{Measured}^{Scaffold}\left( X \right)}{I_{MAX}^{Scaffold}}$ .

A2: Mixed-pixel spatial model

- Model: pixel intensity is a linear combination of background and foreground intensities from the same scaffold channel.

$I_{Measured}^{Scaffold}\left( X \right)=w_{1}*I_{BKG}^{Scaffold}\left( X \right)+w_{2}*I_{FRG}^{Scaffold}\left( X \right)$ *(1)*

$w_{1}+w_{2}=1$ (2)

- The probability of a pixel labeled as scaffold is proportional to the weight of $I_{FRG}^{Scaffold}\left( X \right)$ denoted as $w_{2}$.
- For background equal to the intensities of the dark cross section $I_{BKG}(X)$ and foreground equal to the max intensity $I_{MAX}$, the computation of $w_{2}$ becomes the flat field correction formula:

$w_{2}=\frac{I_{Measured}^{Scaffold}\left( X \right)-I_{BKG}^{Scaffold}\left( X \right)}{{I_{MAX}^{Scaffold}-I}_{BKG}^{Scaffold}\left( X \right)}$ (3)

A3: Mixed-pixel channel model (cell stain bleed through)

- Model: pixel intensity is a linear combination of background and foreground intensities from two channels (cell and scaffold).

$I_{Measured}^{Scaffold}\left( X \right)=w_{1}*I_{BKG}^{Scaffold}\left( X \right)+w_{2}*I_{FRG}^{Scaffold}\left( X \right)+w_{3}*I_{FRG}^{Cell}\left( X \right)$ (4)

$w_{1}+w_{2}+w_{3}=1$ (5)

$w_{2}=\rho*w_{3}$ (6)

- In order to determine the three weights $w_{i}$, we assume that the contribution of scaffold signal is correlated with the cell signal intensity in the last equation and is denoted as $\rho$.
- The probability of a pixel labeled as scaffold is proportional to the weight of $I_{FRG}^{Scaffold}\left( X \right)$ denoted as $w_{2}$.
- For background of scaffold channel equal to the dark cross section $I_{BKG}^{Scaffold}\left( X \right)$, foreground of each channel equal to the max intensity $I_{MAX}^{Cell}$ and $I_{MAX}^{Scaffold}$, the computation of $w_{2}$ becomes:

$w_{2}=\frac{I_{Measured}^{Scaffold}\left( X \right)-I_{BKG}^{Scaffold}\left( X \right)}{I_{MAX}^{Scaffold}+\rho*I_{MAX}^{Cell}-\left( 1-\rho\right)*I_{BKG}^{Scaffold}\left( X \right)}$ (7)

A4: Additive noise model

- Model: pixel intensity consists of ideal signal and additive Gaussian noise

$I_{Measured}^{Scaffold}\left( X \right)=I_{FRG}^{Scaffold}\left( X \right)+N(\mu,\sigma)$ (8)

- The probability of a pixel labeled as scaffold is proportional to $I_{FRG}^{Scaffold}\left( X \right)$ that is obtained by subtracting the Gaussian noise from the measured signal.
- The subtraction is achieved by applying Gaussian filter to $I_{Measured}^{Scaffold}\left( X \right)$ in [X, Y] plane with the filter kernel size equal to 1.06 × s × n^-1/5^, where s is the sample standard deviation estimated from one dark cross section $I_{BKG}^{Scaffold}\left( X \right)$ and n is the sample size [1]. The standard deviation of the Gaussian filter is $\frac{1}{Kernel Size}$.

A5: Markov Random Field (MRF) model

- Model: pixel intensity at X depends on a clique-based correlation among the neighboring pixels from the known clique with $N$ sites.
- Following the notation in [2], the probability of a pixel labeled as scaffold ($L=sc$) (given all image intensities) is proportional to the maximum conditional probability of scaffold labeled pixels inside of a clique $c$ multiplied by the potential function $V_{cL}$of each clique $c$ in a set of all possible cliques $G$.

$P((L=sc)/(I_{Measured}^{Scaffold}\left( X \right)))\propto\prod_{i}^{N} P({(I}_{Measured}^{Scaffold}\left( X_{i} \right))/(L=sc))\prod_{c\in G} e^{{-V}_{cL}}$ (9)

- Using a simple Markov Random Field (MRF) model, interactions can be limited to labels and intensities (hidden and visible variables), cliques in 3D can be defined inside of 3 x 3 x 3 voxel space, and clique potentials can be limited to be positive Boltzman probability distribution functions (PDF) such as $V_{cL}\propto e^{-Energy(Label, Intensity)}$.

**Geometrical models:**

A6: Plane & Vesselness ($\sigma$= 1.0)

- *Spun coat model:* spun coat scaffolds is geometrically modeled by a plane.
- Model evidence: based on experimental measurements of spun coat surfaces using atomic force microscopy (AFM) described in section “Algorithmic model validation measurements”, we used a plane described as $ax+by+cz+d=0$ to approximate the spun coat surfaces.
- Estimation: The plane coefficients were estimated by the constrained least-squared minimization with Lagrange Multipliers method [3] from spun coat voxels per z-stack that have been weighted by normalized voxel intensities to the maximum value.
  - $h=\underset{h}{\mathrm{argmin}} \left\| Ah \right\|$ subject to $\left\| h \right\|=1$,
    where $A=\left[ \begin{matrix} \sqrt{w_{1}}*x_{1} {\sqrt{w_{1}}*y}_{1} {\sqrt{w_{1}}*z}_{1} \\ \vdots\\ \sqrt{w_{N}}*x_{N} {\sqrt{w_{N}}*y}_{N} {\sqrt{w_{N}}*z}_{N} \end{matrix} \right]$, $h={[a,b,c,d]}^{T}$, and $w_{i}$ is the weight at a location [$x_{i}$, $y_{i}$, $z_{i}$].
  - The parameter estimation is achieved when the least-squares error, $h^{T}A^{T}Ah$, is minimum which corresponds to the minimum eigenvalue $\lambda_{i}$ of the matrix $A^{T}A$.
- *Fiber model:* microfiber and medium microfiber scaffolds are geometrically modeled by cylinders.
- Model evidence: based on electrospinning literature [4]–[7], single fibers can be approximated by elastic cylinders.
- Estimation: The local estimation of cylinders extends the Hessian based vessel enhancement filtering (denoted as vesselness or tubeness model) introduced by Frangi et al. [8]. The filtering is based on eigenvalue analysis of the Hessian matrix which allows to extract the principal directions at any location and at multiple scales denoted as $\sigma$. For cylindrical structures, the smallest eigenvalue will correspond to the direction of smallest curvature (along the vessel) while the other two eigenvalues will have a large magnitude of equal sign (+ or – for bright or dark transitions).
- Modified estimation: The modified Frangi’s enhancement filter [8] has been motivated by the lack of fiber vessel enhancement at the fiber crossing in the already published filters [9], [10]. We modified the vessel enhancement filtering as follows:

$I_{Filtered}\left( X \right)=\left( 1-\left| v_{0z} \right| \right)*I_{Measured}(X)$ (10)

where $v_{0z}$ is a z component of the eigenvector with the highest magnitude eigenvalue, $I_{Measured}$ is the original Frangi's vesselness function and $I_{Filtered}$ is the filtering result with enhancements in XY plane.

**
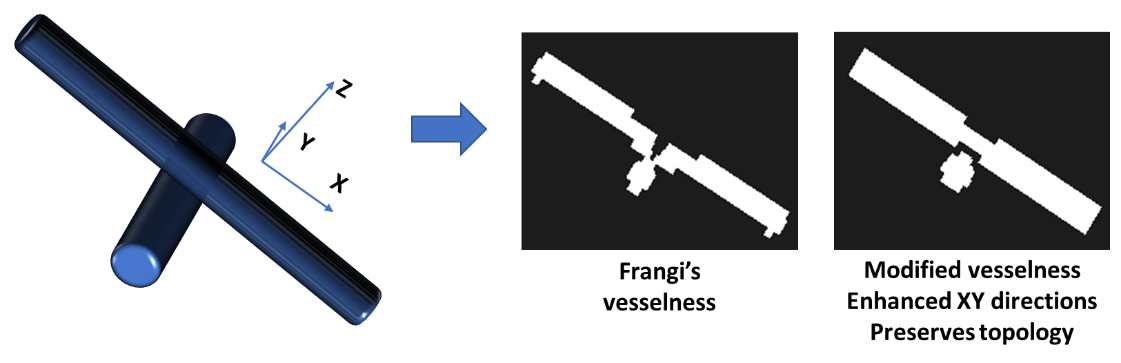
**

(a) (b) (c)

*Figure 1: (a) – Two fiber cylinder models in proximity. (b) - Segmentation results obtained by applying Frangi’s vesselness. (c) – Segmentation obtained by applying modified Frangi’s vesselness. Both (b) and (c) are displayed after thresholding.*

A7: Plane & Vesselness ($\sigma$ =1.5)

- This is the same method as A6 but with a different sigma value for the fiber model. The sigma values were selected to be close to the diameters of the microfibers.

A8: Ad-hoc Thresholding and Gaussian Filtering

- Model: manual choice of segmentation steps and their parameters according to visual inspection of z-stack sample files performs satisfactorily over a large collection of z-stacks.
- Estimation: Gaussian filtering followed by thresholding to achieve z-stack segmentation. The sigma value of Gaussian kernel was determined based on visual inspection and with the intention to reduce noise and avoid an excessive image blur.

After either computing probability or applying the vessel enhancement filtering, the cropped scaffold z-stacks are masked by the verified cell segmentation and the values are adaptively thresholded using maximum entropy criterion [11] to obtain binary scaffold segmentation.

**References**

[1] L.-C. Chang, G. K. Rohde, and C. Pierpaoli, “An automatic method for estimating noise-induced signal variance in magnitude-reconstructed magnetic resonance images,” *Med. Imaging. Int. Soc. Opt. Photonics*, pp. 1136–1142, 2005.

[2] M. Berthod, Z. Kato, S. Yu, and J. Zerubia, “Bayesian image classification using Markov random fields,” *Image Vis. Comput.*, vol. 14, no. 4, pp. 285–295, 1996.

[3] I. Griva, S. G. Nash, and A. Sofer, *Linear and Nonlinear Optimization*, 2nd ed. SIAM, 2009.

[4] M. Lauricella, G. Pontrelli, I. Coluzza, D. Pisignano, and S. Succi, “JETSPIN: A specific-purpose open-source software for simulations of nanofiber electrospinning,” *Comput. Phys. Commun.*, vol. 197, pp. 227–238, 2015.

[5] A. L. Yarin, S. Koombhongse, and D. H. Reneker, “Bending instability in electrospinning of nanofibers,” *J. Appl. Phys.*, vol. 89, no. 5, pp. 3018–3026, 2001.

[6] S. Chew, Y. Wen, Y. Dzenis, and K. Leong, “The Role of Electrospinning in the Emerging Field of Nanomedicine,” *Curr. Pharm. Des.*, vol. 12, no. 36, pp. 4751–4770, 2006.

[7] A. S. Nain, M. Sitti, A. Jacobson, T. Kowalewski, and C. Amon, “Dry spinning based spinneret based tunable engineered parameters (STEP) technique for controlled and aligned deposition of polymeric nanofibers,” *Macromol. Rapid Commun.*, vol. 30, no. 16, pp. 1406–1412, 2009.

[8] A. F. Frangi, W. J. Niessen, K. L. Vincken, and M. a Viergever, “Multiscale vessel enhancement filtering,” *Medial Image Comput. Comput. Invervention - MICCAI’98. Lect. Notes Comput. Sci. vol 1496*, vol. 1496, pp. 130–137, 1998.

[9] M. Erdt, M. Raspe, and M. Suehling, “Automatic hepatic vessel segmentation using graphics hardware,” in *Medical Imaging and Virtual Reality*, Lecture No., T. Dohi, I. Sakuma, and H. Liao, Eds. Tokyo, Japan: Springer Berlin Heidelberg, 2008, pp. 403–412.

[10] Y. Sato *et al.*, “3D multi-scale line filter for segmentation and visualization of curvilinear structures in medical images,” in *CVRMed-MRCAS’97: First Joint Conference Computer Vision, Virtual Reality and Robotics in Medicine and Medical Robotics and Computer-Assisted Surgery Grenoble, France, March 19--22, 1997 Proceedings*, Springer Berlin Heidelberg, 1997, pp. 213--222.

[11] M. Sezgin and B. Sankur, “Survey over image thresholding techniques and quantitative performance evaluation,” *J. Electron. Imaging*, vol. 13, no. 1, pp. 146–165, 2004.
