# Supplementary material for: Modeling, validation and verification of three-dimensional cell-scaffold contacts from terabyte-sized images
Source: BMC Bioinformatics. 2017 Nov 28;18:526. doi: 10.1186/s12859-017-1928-x (PMC5706418; doi:10.1186/s12859-017-1928-x)
Supplement: Supplementary file 6 — Algorithms based on statistical models for segmenting all scaffold types. (DOCX 48 kb) [file 12859_2017_1928_MOESM6_ESM.docx]

# Additional file 6: Algorithms based on statistical models for segmenting all scaffold types

A1: Single-pixel model

1. Find a dark cross-section (first or last slice in a z-stack) and compute intensity histogram of the dark cross-section.
2. Determine background threshold per channel such that it satisfies $P\left( I>I_{Thresh} \right)=5 \%$ based on the computed histogram.
3. Assign label BKG and FRG to pixels in each channel based on the threshold value per channel.
4. Find max intensity value in each FRG pixel set.
5. Compute probability of assigning a label at a pixel level (cell or scaffold labels) as $\frac{I(X)}{I_{MAX}}$ per channel.
6. Report label and probability images.

## A2: Mixed-pixel spatial model

1. Find a dark cross-section, $I_{BKG}^{Scaffold}\left( X \right)$.
2. Apply the flat field correction formula to obtain $w_{2}$ at all $X$ as follows.

$w_{2}(X)=\frac{I_{Measured}^{Scaffold}\left( X \right)-I_{BKG}^{Scaffold}\left( X \right)}{{I_{MAX}^{Scaffold}-I}_{BKG}^{Scaffold}\left( X \right)}$ (16)

1. Compute probability of assigning a label at a voxel $X$ as $P\left( X \right)=w_{2}(X)/max\{w_{2}\}$ per channel.
   1. Set any pixels such that $w_{2}<0$ to 0; check if $\max\left\{ w_{2} \right\}=1$.
2. Assign label BKG and FRG to pixels in each channel based on the threshold value per channel ($th=0.5$).
3. Report label and probability images.

## A3: Mixed-pixel channel model

**Algorithmic sequence for cell channel would be similar to the scaffold channel described below:**

1. Find a dark cross-section, $I_{BKG}^{Scaffold}\left( X \right)$.
2. Determine background threshold per channel such that it satisfies $P\left( I>I_{Thresh} \right)=5 \%$based on the histogram of the dark cross section.
3. Estimate the correlation coefficient ρ between cell channel pixels and scaffold channel pixels that satisfy $I_{Measured}^{Scaffold}\left( X \right)>I_{Thresh}$.
4. Apply the mixed pixel formula

$w_{2}(X)=\frac{I_{Measured}^{Scaffold}\left( X \right)-I_{BKG}^{Scaffold}\left( X \right)}{I_{MAX}^{Scaffold}+\rho*I_{MAX}^{Cell}-\left( 1-\rho\right)*I_{BKG}^{Scaffold}\left( X \right)}$ (17)

to obtain $w_{2}(X)$.

1. Compute probability of assigning a scaffold label at a voxel $X$ as $P\left( X \right)=w_{2}(X)/max\{w_{2}\}$ per channel.
2. Assign label BKG and FRG to pixels in scaffold channel based on the threshold value ($th=0.5$).
3. Report label and probability images for scaffold channel.

## A4: Additive noise models (with spatial extend) for an ideal single-pixel signal

1. Find a dark cross-section and compute the sample mean and standard deviation of the dark cross-section, i.e., $\mu\left\{ I_{BKG}^{Scaffold}\left( X \right) \right\}, \sigma\left\{ I_{BKG}^{Scaffold}\left( X \right) \right\}$.
2. Apply Gaussian filter to $I_{Measured}^{Scaffold}\left( X \right)$ in 2D with $\mathrm{kernel}_{Size}=1.06 \times s \times n^{-1/5}$, where $s$ is the sample standard deviation and $n$ is the sample size; standard deviation of the kernel $\mathrm{kernel}_{Stdev}=1/\mathrm{kernel}_{Size}$.

$I_{Gauss}^{Scaffold}=I_{Measured}^{Scaffold}\left( X \right)\circ G(-\mu\left\{ I_{BKG}^{Scaffold}\left( X \right) \right\},\mathrm{kernel}_{Stdev},\mathrm{kernel}_{Size})$ (18)

1. Compute probability of assigning a label at a voxel $X$ as $P\left( X \right)=\left| I_{Gauss}^{Scaffold}(X) \right|/max\left\{ I_{Gauss}^{Scaffold}(X) \right\}$ per channel.
2. Determine background threshold per channel such that it satisfies $P\left( I_{Gauss}^{Scaffold}(X)>I_{Thresh} \right)=5 \%$based on the histogram of dark cross section after filtering.
3. Assign label BKG and FRG to pixels in each channel based on the threshold value per channel and the computed probabilities.
4. Report label and probability images.

## A5: Markov Random Field (MRF) model of clique-based correlation among the neighboring pixels

**Algorithmic sequence (deviates from simulated annealing and follows worst-best case scenario):**

1. Find a dark cross-section and compute intensity histogram of the dark cross-section.
2. Determine background threshold per channel such that it satisfies $P\left( I>I_{Thresh} \right)=5 \%$ based on the computed histogram.
3. Apply morphological dilation to $I_{Measured}^{Scaffold}\left( X \right)$ in 3D using 3x3x3 kernel to obtain $I_{dilated}^{Scaffold}\left( X \right)$.
4. Apply morphological erosion to $I_{Measured}^{Scaffold}\left( X \right)$ in 3D using 3x3x3 kernel to obtain $I_{eroded}^{Scaffold}\left( X \right)$.
5. Assign label BKG and FRG to pixels in each channel based on the threshold value per channel according to the following rules.
   1. If ${I_{Thresh}<I}_{eroded}^{Scaffold}\left( X \right)$ then assign FRG.
   2. If ${I_{Thresh}>I}_{dilated}^{Scaffold}\left( X \right)$ then assign BKG.
   3. If $I_{eroded}^{Scaffold}\left( X \right)<{I_{Thresh}<I}_{dilated}^{Scaffold}\left( X \right)$ & $(I_{Measured}^{Scaffold}\left( X \right) - I_{eroded}^{Scaffold}\left( X \right))/(I_{dilated}^{Scaffold}\left( X \right)-I_{eroded}^{Scaffold}\left( X \right))>0.5$ & $( {(I}_{Thresh}-I_{eroded}^{Scaffold}\left( X \right))/{{(I}_{Thresh}-I_{eroded}^{Scaffold}\left( X \right))/(I}_{dilated}^{Scaffold}\left( X \right)-I_{eroded}^{Scaffold}\left( X \right))<0.5$ then assign FRG else BKG
   4. Assign probability to each pixel as $0.5*\left( I_{dilated}^{Scaffold}\left( X \right)+I_{eroded}^{Scaffold}\left( X \right) \right)/max\left\{ I_{Measured}^{Scaffold}\left( X \right) \right\}$.
6. Report label and probability images.

The source code can be found at <https://github.com/usnistgov/cell-scaffold-contact>.
