# Supplementary material for: Modeling, validation and verification of three-dimensional cell-scaffold contacts from terabyte-sized images
Source: BMC Bioinformatics. 2017 Nov 28;18:526. doi: 10.1186/s12859-017-1928-x (PMC5706418; doi:10.1186/s12859-017-1928-x)
Supplement: Supplementary file 7 — Algorithm based on planar geometrical model for segmenting spun coat scaffolds. (DOCX 36 kb) [file 12859_2017_1928_MOESM7_ESM.docx]

# Additional file 7: Algorithm based on planar geometrical model for segmenting spun coat scaffolds

1. Define a plane model: The plane model was defined as $f\left( x,y,z \right)=ax+by+cz+d$.
2. Solve a weighted least-squares problem: The solution can be found by solving the constrained least-squares minimization problem with Lagrange multipliers^[[1]](#footnote-1)^ [1], which yields the eigenvector corresponding to the smallest eigenvalue of $X^{T}WX$, where $X=\left[ \begin{matrix} x_{1} \\ \vdots\\ x_{N} \end{matrix} \begin{matrix} y_{1} \\ \vdots\\ y_{N} \end{matrix} \begin{matrix} z_{1} \\ \vdots\\ z_{N} \end{matrix} \begin{matrix} 1 \\ \vdots\\ 1 \end{matrix} \right]$, $W=\left[ \begin{matrix} w_{1} & 0 & 0 \\ 0 & \ddots& 0 \\ 0 & 0 & w_{N} \end{matrix} \right]$, $w_{i}$ is the weight for a voxel at a location [$x_{i}$, $y_{i}$, $z_{i}$] in a scaffold z-stack ($i=1,2,\ldots, N$) and is given as ${I(x_{i},y_{i},z_{i})}^{4}$, $I(x_{i},y_{i},z_{i})$ is the normalized intensity value at [$x_{i}$, $y_{i}$, $z_{i}$], and $N$ is the total number of voxels in the scaffold z-stack.
3. Estimate upper bound plane and lower bound plane of the spun coat: (i) A new coordinate system (X’Y’Z’) is obtained, such that the X’Y’X’Y’-plane maps to the estimated plane in Step 2 and the Z’Z’-axis corresponds to the normal vector of the estimated plane. (ii) All voxel values are projected onto Y’Z’Y’Z’-plane and averaged at all $(y^{'},z^{'})$.$)$ (iii) The lower and upper bounds of the spun coat are estimated by analyzing the Z-profile the same way as for cropping (Additional file 3).
4. Compute the residual standard deviation and pooled standard deviation.

The source code can be found at <https://github.com/usnistgov/cell-scaffold-contact>.

References

1. Griva I, Nash SG, Sofer A. Linear and Nonlinear Optimization. 2nd ed. SIAM; 2009.

1. https://www.mathworks.com/help/optim/ug/lsqlin.html [↑](#footnote-ref-1)
