# Supplementary material for: Modeling, validation and verification of three-dimensional cell-scaffold contacts from terabyte-sized images
Source: BMC Bioinformatics. 2017 Nov 28;18:526. doi: 10.1186/s12859-017-1928-x (PMC5706418; doi:10.1186/s12859-017-1928-x)
Supplement: Supplementary file 8 — Algorithms based on cylindrical geometrical models for segmenting fiber scaffolds. (DOCX 25 kb) [file 12859_2017_1928_MOESM8_ESM.docx]

# Additional file 8: Algorithms based on cylindrical geometrical models for segmenting fiber scaffolds

## A6*: Modified Frangi’s vesselness (Frangi F10)

1. Frangi’s vesselness computation according to [37]; ($\sigma$ = 1.0, C = 1, $\alpha$ = 0.5, $\beta$ = 0.5).
2. Image min-max normalization in range of 0 to 255.
3. Gray-scale thresholding at the level of 20/256 (≈8 %).
4. White object hole filling.
5. Skeletonization and center-line tracking.
6. Diameter estimation and 3D modeling.

## A7*: Modified Frangi’s vesselness (Frangi F15)

The same as A6* but using $\sigma$ = 1.5.

**Note: The** $\sigma$ parameterin the A6 and A7 models determines radii of fibers to be enhanced. Diameters of fibers with much smaller or much higher radii compared to $\sigma$will be incorrectly estimated. The asterisk next to A6 and A7 indicates that we used modified Frangi’s vesselness.

## A8: Ad-hoc Thresholding and Gaussian Filtering

1. Image min-max normalization in range of 0 to 255.
2. Gray-scale thresholding at the level of 20/256.
3. Gaussian kernel filtering ($\sigma$ = 0.3).
4. Gray-scale thresholding at the level of 128/256.
5. Steps 5 to 9 are the same as steps 2 to 6 in A6* algorithm.

The source code can be found at <https://gitlab.com/piotr.szczypinski/microfibers>.
