# Supplementary material for: Modeling, validation and verification of three-dimensional cell-scaffold contacts from terabyte-sized images
Source: BMC Bioinformatics. 2017 Nov 28;18:526. doi: 10.1186/s12859-017-1928-x (PMC5706418; doi:10.1186/s12859-017-1928-x)
Supplement: Supplementary file 9 — Evaluation of goodness-of-fit for planar model used for modeling spun coat scaffolds. (DOCX 796 kb) [file 12859_2017_1928_MOESM9_ESM.docx]

# Additional file 9: Evaluation of goodness-of-fit for planar model used for modeling spun coat scaffolds

Geometrical modeling of spun coat scaffolds as planar surfaces was presented in Section “Geometrical modeling: Cell-scaffold contact from 3 Methods”. We sorted and plotted all residual standard deviations of a planar fit for each SC z-stack in Figure 1. This figure shows the distribution of residual standard deviations across 165 z-stacks of SC scaffolds. We also computed the average thickness (upper-lower plane difference) to be 4.738 µm ±1.348 µm with minimum 2.310 µm and maximum of 12.012 µm.

Figure 2(a) shows some variability in slopes of spun coat with respect to the Z-axis which indicates not all spun coat scaffolds are perpendicularly aligned to the z-axis. We found some local curvatures of spun coat (see Figure 2(b)) that could become a source of error in contact point estimation using geometrical models.


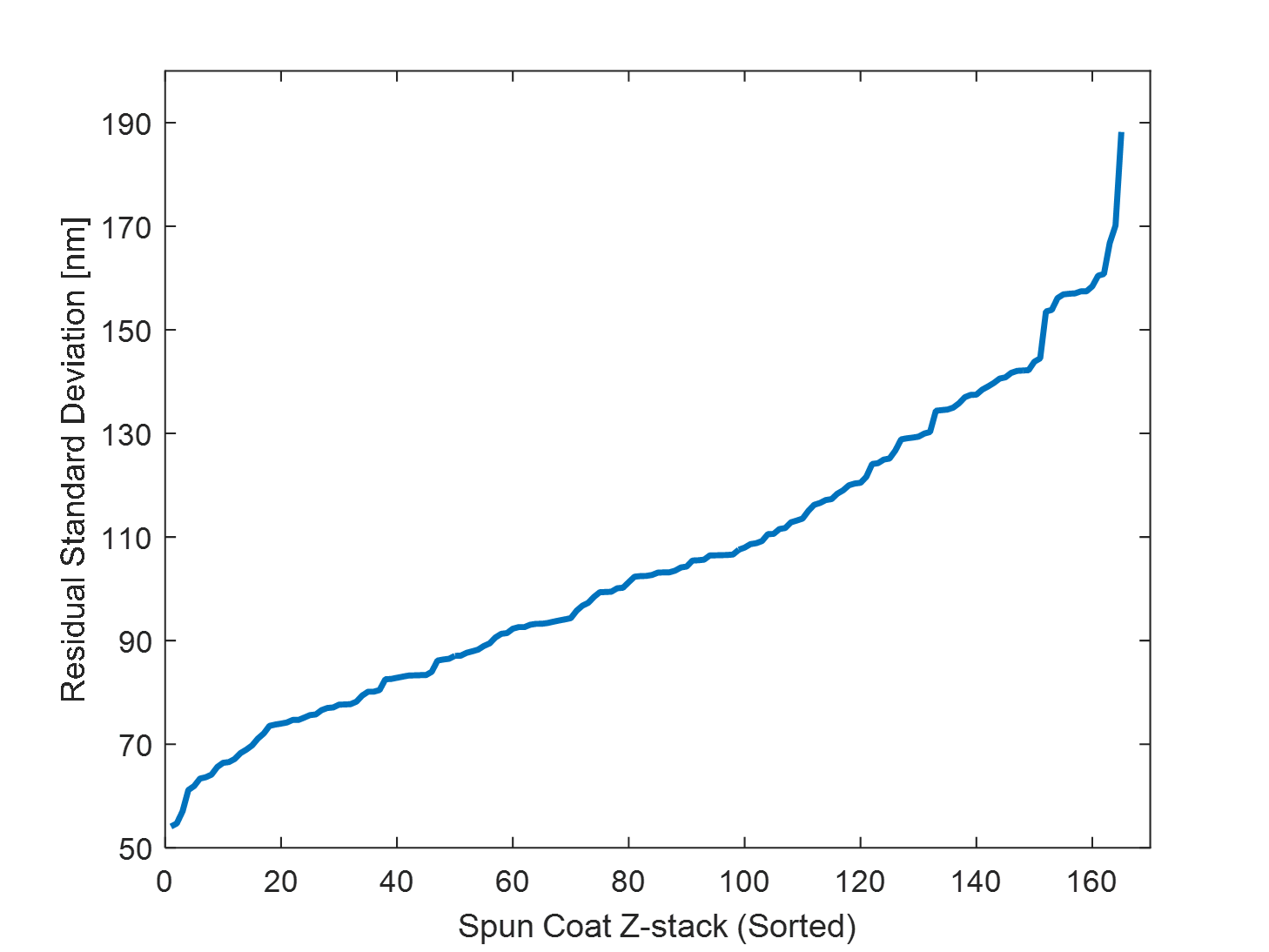


Figure 1: Sorted residual standard deviations of a planar fit to 165 spun coat z-stacks


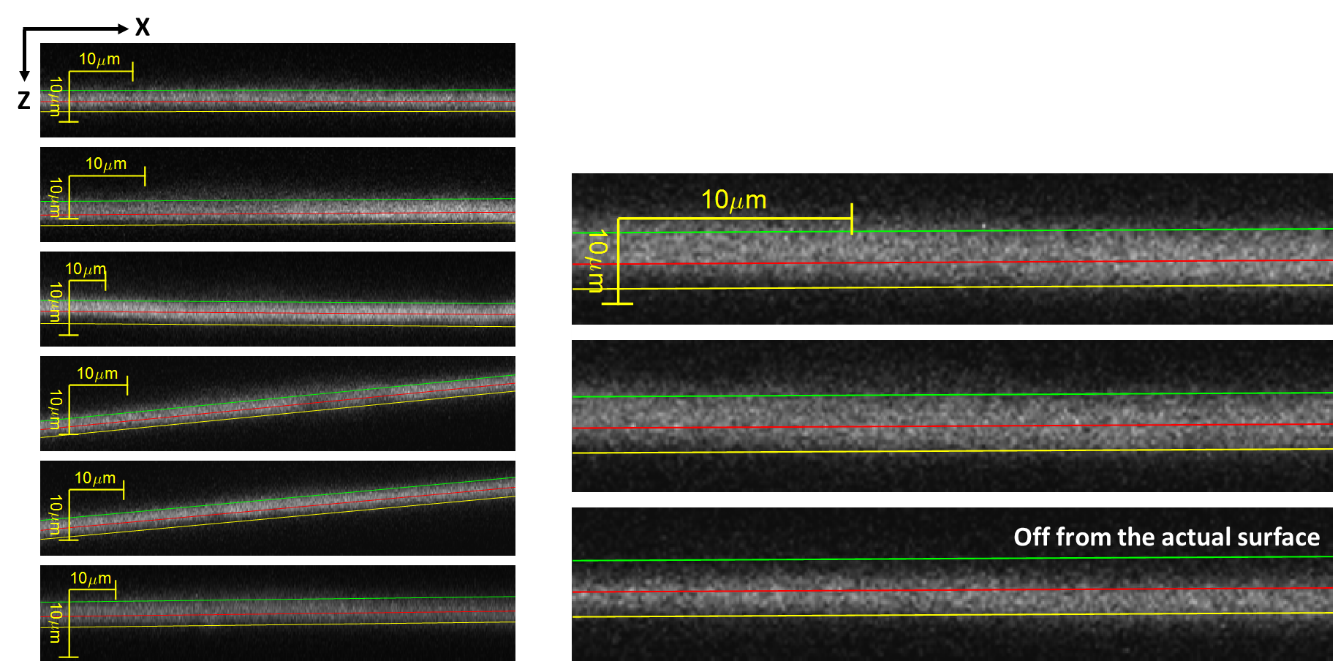


(a) (b)

Figure 2: (a) – [X, Z] cross-sections of 6 spun coat z-stacks after plane fitting (red line) with lower Z (green) and higher Z (yellow) parallel boundaries. (b) - [X, Z] cross-sections of one spun coat z-stack at multiple Y locations after plane fitting to illustrate minor curvature and thickness change.
