# Supplementary material for: Modeling, validation and verification of three-dimensional cell-scaffold contacts from terabyte-sized images
Source: BMC Bioinformatics. 2017 Nov 28;18:526. doi: 10.1186/s12859-017-1928-x (PMC5706418; doi:10.1186/s12859-017-1928-x)
Supplement: Supplementary file 10 — Validation steps based on 2D SEM and 3D CLSM data of Single Fibers. (DOCX 34 kb) [file 12859_2017_1928_MOESM10_ESM.docx]

Additional file 10: Validation steps based on 2D SEM and 3D CLSM data of Single Fibers

The step indices refer to Fig. 10 in the main manuscript.

- Step 0: establish reference diameter measurements from 2D SEM images.
  - Segment images using ImageJ/Fiji.
  - Skeletonize segments using ImageJ/Fiji.
  - Estimate diameters at the skeleton points using DiameterJ plugin to ImageJ.
  - Visually verify the accuracy of diameters from perpendicular and 65 degree views of fibers in 2D SEM image segmentations (accuracy estimated to be around 3 %).
- Step 1: segment single fiber CLSM z-stacks using the designed algorithms applied to experimental CLSM z-stacks of scaffolds.
- Step 2: extract skeleton and diameter from segmented CLSM z-stacks using VesselKnife software accessible from <https://gitlab.com/vesselknife/vesselknife>.
  - Apply median filter to remove floating single voxels and fill small holes in data using the ITK implementation (kernel radius=2 corresponds to 5x5 kernel).
  - Fill holes using the ITK implementation (no parameter – Flood Filling Alg.).
  - Apply binary skeletonization using the ITK implementation (no parameters).
  - Extract the diameter at each skeleton point as the smaller eigenvalue of a covariance matrix computed from all point coordinates defined by 12 outgoing rays toward vertices of a convex regular icosahedron.
- Step 3: identify reference piece-wise linear segments in each z-stack that match the fiber that has been measured in SEM (manual process).
- Step 4: select registration points in z-stacks that are within a small neighborhood of the reference linear segments.
- Step 5: estimate pair-wise vertical translation vectors to stitch 16 CLSM z-stacks.
- Step 6: select two sets of skeleton points from 16 FOVs: all points and points from non-overlapping volumes of z-stacks.
- Step 7: compute the average and standard deviation of diameters over the two sets of skeleton points, as well as their histograms.

**Note:** One of the unresolved challenges in fiber scaffold manufacturing is the unknown geometrical placement of fibers. As of today, we cannot control the scaffold geometry which limits the validation of cell-fiber scaffold contact measurements. For validation, we considered specific-purpose open-source software for simulations of nanofiber electrospinning called JetSpin<http://www.nanojets.eu/downloads.html>. However, it is not trivial to map experimental settings of our electrospun device and variations of the material and the environment into parameters and assumptions of the JetSpin simulation of nanofiber electrospinning. Thus, we focus on single fiber measurements and extraction of the fiber radii along the fiber. The drawbacks of the single fiber approach are that it applies only to microfibers, fiber sampling is ad-hoc, and fiber to fiber interaction might not be fully captured in single fiber measurements. We are also missing a direct point-to-point relationship between SEM and fluorescent CLSM radius measurements and could not assess how cell-scaffold channel bleed-through affects the microfiber measurements. We also had to resolve a fiber swelling issue by expedited measurements since the SEM measurements are taken from a dry sample while the fluorescent CLSM measurements are taken from a sample in water.
